# T follicular helper cells regulate the activation of B lymphocytes and antibody production during *Plasmodium vivax* infection

**DOI:** 10.1371/journal.ppat.1006484

**Published:** 2017-07-10

**Authors:** Maria Marta Figueiredo, Pedro Augusto Carvalho Costa, Suelen Queiroz Diniz, Priscilla Miranda Henriques, Flora Satiko Kano, Mauro Sugiro Tada, Dhelio Batista Pereira, Irene Silva Soares, Olindo Assis Martins-Filho, Dragana Jankovic, Ricardo Tostes Gazzinelli, Lis Ribeiro do Valle Antonelli

**Affiliations:** 1 Laboratório de Biologia e Imunologia de Doenças Infecciosas e Parasitárias, Instituto René Rachou, Fundação Oswaldo Cruz, Belo Horizonte, Minas Gerais, Brazil; 2 Laboratório de Imunopatologia, Instituto René Rachou, Fundação Oswaldo Cruz, Belo Horizonte, Minas Gerais, Brazil; 3 Departamento de Bioquímica e Imunologia, Universidade Federal de Minas Gerais, Belo Horizonte, Minas Gerais, Brazil; 4 Laboratório de Malária, Instituto René Rachou, Fundação Oswaldo Cruz, Belo Horizonte, Minas Gerais, Brazil; 5 Centro de Pesquisas em Medicina Tropical de Rondônia, Porto Velho, Rondônia, Brazil; 6 Departamento de Análises Clínicas e Toxicológicas, Faculdade de Ciências Farmacêuticas, Universidade de São Paulo, São Paulo, São Paulo, Brazil; 7 Laboratório de Biomarcadores de Diagnóstico e Monitoração, Instituto René Rachou, Fundação Oswaldo Cruz, Belo Horizonte, Minas Gerais, Brazil; 8 Immunobiology Section, Laboratory of Parasitic Diseases, National Institute of Allergy and Infectious Diseases, National Institutes of Health, Bethesda, Maryland, United States of America; Queensland Institute of Medical Research, AUSTRALIA

## Abstract

Although the importance of humoral immunity to malaria has been established, factors that control antibody production are poorly understood. Follicular helper T cells (Tfh cells) are pivotal for generating high-affinity, long-lived antibody responses. While it has been proposed that expansion of antigen-specific Tfh cells, interleukin (IL) 21 production and robust germinal center formation are associated with protection against malaria in mice, whether Tfh cells are found during *Plasmodium vivax* (*P*. *vivax*) infection and if they play a role during disease remains unknown. Our goal was to define the role of Tfh cells during *P*. *vivax* malaria. We demonstrate that *P*. *vivax* infection triggers IL-21 production and an increase in Tfh cells (PD-1+ICOS+CXCR5+CD45RO+CD4+CD3+). As expected, FACS-sorted Tfh cells, the primary source of IL-21, induced immunoglobulin production by purified naïve B cells. Furthermore, we found that *P*. *vivax* infection alters the B cell compartment and these alterations were dependent on the number of previous infections. First exposure leads to increased proportions of activated and atypical memory B cells and decreased frequencies of classical memory B cells, whereas patients that experienced multiple episodes displayed lower proportions of atypical B cells and higher frequencies of classical memory B cells. Despite the limited sample size, but consistent with the latter finding, the data suggest that patients who had more than five infections harbored more Tfh cells and produce more specific antibodies. *P*. *vivax* infection triggers IL-21 production by Tfh that impact B cell responses in humans.

## Author summary

*Plasmodium vivax* is the most widely spread malaria parasite species and represents a significant impediment to social and economic development in endemic countries. Our goal was to assess the importance of T follicular helper cells in the development of the immune response during malaria. We found that *P*. *vivax* infection promotes expansion of circulating Tfh cells that secrete IL-21 to boost immunoglobulin production by B-cells. Accordingly, malaria infection led to marked changes in B cell subpopulations, including expansion of plasma cells and increased production of antigen-specific IgG1 and IgG3. Re-exposure to *P*. *vivax* led to amplified Tfh cells cell responses that were concomitantly associated with increased frequencies of classical memory B cells. Thus, Tfh cells that are induced during *P*. *vivax* infection could impact the efficiency of humoral immune responses that underlie protective immunity.

## Introduction

Malaria, caused by the protozoan parasite *Plasmodium*, remains one of the most widespread and mortality-causing infectious diseases worldwide. *Plasmodium vivax* is the most frequent cause of recurring malaria and infects 130–390 million people each year, representing approximately 50% of all malaria cases [1]. Through constant reinfection, adult individuals acquire clinical immunity against severe disease by controlling infection, regardless of the parasite species. These individuals can become asymptomatic parasite carriers of both asexual blood-stage and infective sexual gametocyte stages [2]. Clinical immunity depends on antibodies [3], however it is assumed that protective humoral responses to malaria are short-lived, slowly develop after multiple exposures to parasites and can be lost in the absence of regular exposure [4].

In addition to the clinical amelioration, resolution of malaria depends on generation of pathogen-specific antibodies. T follicular helper cells (Tfh cells) are key orchestrators of the germinal center (GC) reactions that drive the generation of plasma cells that secrete high-affinity antibodies to resolve primary infection and long-lived memory B cells that maintain protection against re-infection [5]. Tfh cells can be distinguished from other Th populations based on anatomical localization, effector functions, development requirements and homing properties [6]. Tfh cells priming is driven by cognate interaction between naive CD4^+^ T cells and conventional dendritic cells producing IL-6 and IL-21 that induce B-cell lymphoma 6 protein (Bcl-6), a transcriptional repressor promoting expression of C-X-C chemokine receptor type 5 (CXCR5). CXCR5 endows lymphocytes with the capacity to migrate to B cell follicles rich in C-X-C motif chemokine ligand 13 (CXCL13) [7,8]. Tfh cells motility is also regulated by ICOS-ICOS-L (Inducible T-cell Costimulator) interactions between Tfh cells and non-cognate B cells at the T-B border, which potentiates Tfh cells migration into the follicle [9]. Once in the follicle, Tfh cells activity depends on cognate interactions with B cells, which further reinforces Tfh cells differentiation and function [9,10]. Therefore, their unique phenotype is critical for their development and function [7,9,11,12,13]. Cytokine production triggered by microbes at the onset of infection can also influence Tfh cell development [14]. Indeed, the absence of IL-21 results in reduced antibody production and in decreased GC B cell numbers that correlate with to a profound defect in GC formation [15].

In human blood, CXCR5^+^CD4^+^ T cells display Tfh cell functional properties, including being able to efficiently induce naïve B cells to produce immunoglobulin via IL-21 secretion, and are believed to represent the circulating memory counterpart of the Tfh cells from lymphoid tissues [11].

Protection in an experimental malaria vaccination protocol was associated with enhanced expansion of antigen-specific Tfh cells and robust GC formation [16]. Moreover, the absence of IL-21, produced by T cells, abrogates *P*. *chabaudi*-specific immunoglobulin secretion and memory B cell responses [17]. In addition, in mice, severe malaria induces impaired Tfh cells differentiation and defective germinal centers. In this case, despite IL-21 production, Tfh cells expressed low levels of programmed cell death protein 1 (PD-1) and CXCR5 and co-expressed Th1-associated molecules [18]. Currently, there is only one published study assessing Tfh cells in humans infected with *P*. *falciparum*, which showed that while malaria drives Th1 cytokine responses and Th1-like Tfh cells, their activation status did not correlate with antibody production [19].

Given the importance of T cell-dependent antibody responses in malaria, we attempted to assess circulating Tfh cells and define their role during *P*. *vivax* infection. To address this issue, we phenotypically and functionally characterized T and B cell subsets in the peripheral blood from patients experiencing acute malaria episodes. We demonstrate that *P*. *vivax* infection triggers an increase in circulating Tfh cells during acute infection and that Tfh cells are the primary sources of IL-21 and induce immunoglobulin production by naïve B cells. Moreover, *P*. *vivax* malaria alters the B cell compartments and these alterations are dependent on the number of malaria re-infections. Taken together, our findings indicate that circulating Tfh cells may be a marker of humoral responses against *Plasmodium* infection in humans.

## Results

### Study subjects

The malaria group consisted of three females (12.5%) and 21 males (87.5%) with an age range from 18 to 56 years (median 33.61 ± SD 8.88 years old). In Brazil, malaria is an occupational disease and therefore affects mostly males. Thirty three percent asserted primary malaria infection, 38% reported 1–5 previous malaria episodes and 29% reported more than five previous malaria episodes. Numbers of malaria episodes were confirmed by electronic records obtained from the Ministry of Health (Sivep). All patients presented clinical symptoms of malaria and *P*. *vivax* parasitaemia ranged from 2.24 to 12,641.45 (median 18.39 ± SD 3.281) parasites/μL (S1 Table). Peripheral blood mononuclear cells (PBMC) viability after thawing, analyzed by flow cytometry using Acqua or Violet Live/Dead (Invitrogen), was similar between malaria patients (median 82.21 ± SD 13.26) and healthy individuals (median 86.02 ± SD 5.094). Healthy donors included in the studies were from the same endemic area and had not had any malaria episode by the date the blood samples were collected. They did not present with any other symptoms and were not on medication for any chronic disease.

### *P*. *vivax* triggers the expression of activation markers on T cell subsets

**Fig 1:**
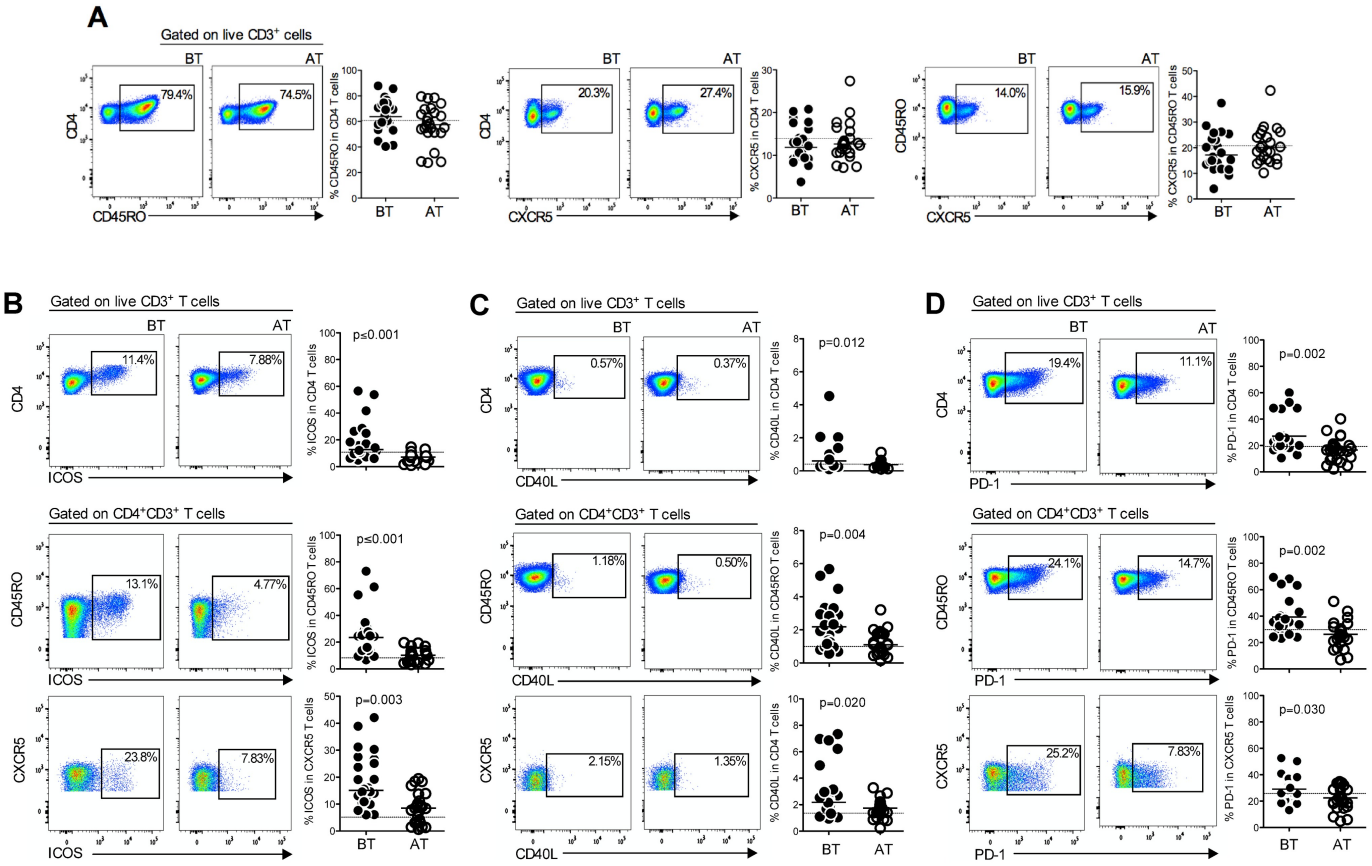
*Plasmodium vivax* infection triggers the expression of activation markers on CD4^+^ T, CD45RO^+^CD4^+^ T and CXCR5^+^CD4^+^ T cells. PBMC from *P*. *vivax*-infected patients were analyzed ex vivo (n = 24) during acute malaria episode (BT, filled circles) and 30–45 days after treatment (AT, open circles). All the T cell subsets were analyzed after gating on live CD3^+^ cells. **A**. Representative density plots showing frequency of CD4^+^, CD45RO^+^CD4^+^, and CXCR5^+^CD45RO^+^CD4^+^ T cells (left to right) from a single *P*. *vivax*-infected patient. Scattered plots showing the percentage of CD4^+^ T cells and CD45RO within CD4^+^ T cells, and CXCR5 within CD45RO^+^CD4^+^ T lymphocytes in *P*. *vivax*–infected patients measured before and after treatment (left to right). **B-D**. Representative density plots showing frequency of ICOS (B), CD40L (C) and PD-1 (D) expressing CD4^+^, CD45RO^+^CD4^+^, and CXCR5^+^ CD4^+^ T cells (top to bottom) from a single *P*. *vivax*-infected patient (left panels). Frequency of ICOS (B), CD40L (C) and PD-1 (D) within the T cell subsets described (right panels). Lines represent median values of the given measurement in each group. Dotted lines represent healthy donors. *p* values are depicted in the figure.

Studies on healthy adults have shown that blood CXCR5^+^CD4^+^ T cells are the circulating counterparts of GC Tfh cells in secondary lymphoid tissue [11]. We investigated the expression of molecules expressed by activated and memory cells, and those that define Tfh cells, in PBMC from *P*. *vivax*-infected patients before treatment (BT) and after treatment with chloroquine and primaquine (AT). As an additional control, we analyzed PBMC from healthy donors (HD). No alterations were found in the frequencies of memory (CD45RO^+^), activated (CXCR5^+^) and activated memory (CXCR5^+^CD45RO^+^) CD4^+^ T cells during acute malaria when comparing the same patients BT and AT (Fig 1A). However, we found that the frequency of ICOS expressing cells was significantly increased in the memory, activated memory and total CD4^+^ T lymphocyte compartments in malaria patients BT (Fig 1B). However, AT ICOS expression returned to the levels observed on CD4^+^ T cells from healthy donors (HD) (Fig 1B, S3 Table). Furthermore, we observed increased frequencies of CD40L^+^ cells among CD45RO^+^, CXCR5^+^CD45RO^+^ and total CD4^+^ T cells during acute malaria (Fig 1C). Moreover, acute *P*. *vivax* infection also triggered the expression of PD-1, another member of the B7-CD28 family [20], on CD4^+^, CD45RO^+^CD4^+^ and CXCR5^+^CD45RO^+^CD4^+^ T cells (Fig 1D). Taken together, our data demonstrate that ICOS, CD40L and PD-1 are upregulated by CD4^+^ T cells during *P*. *vivax* infection.

### *P*. *vivax* infection promotes an expansion of circulating Tfh cells and increased levels of Tfh cells-related cytokines

**Fig 2:**
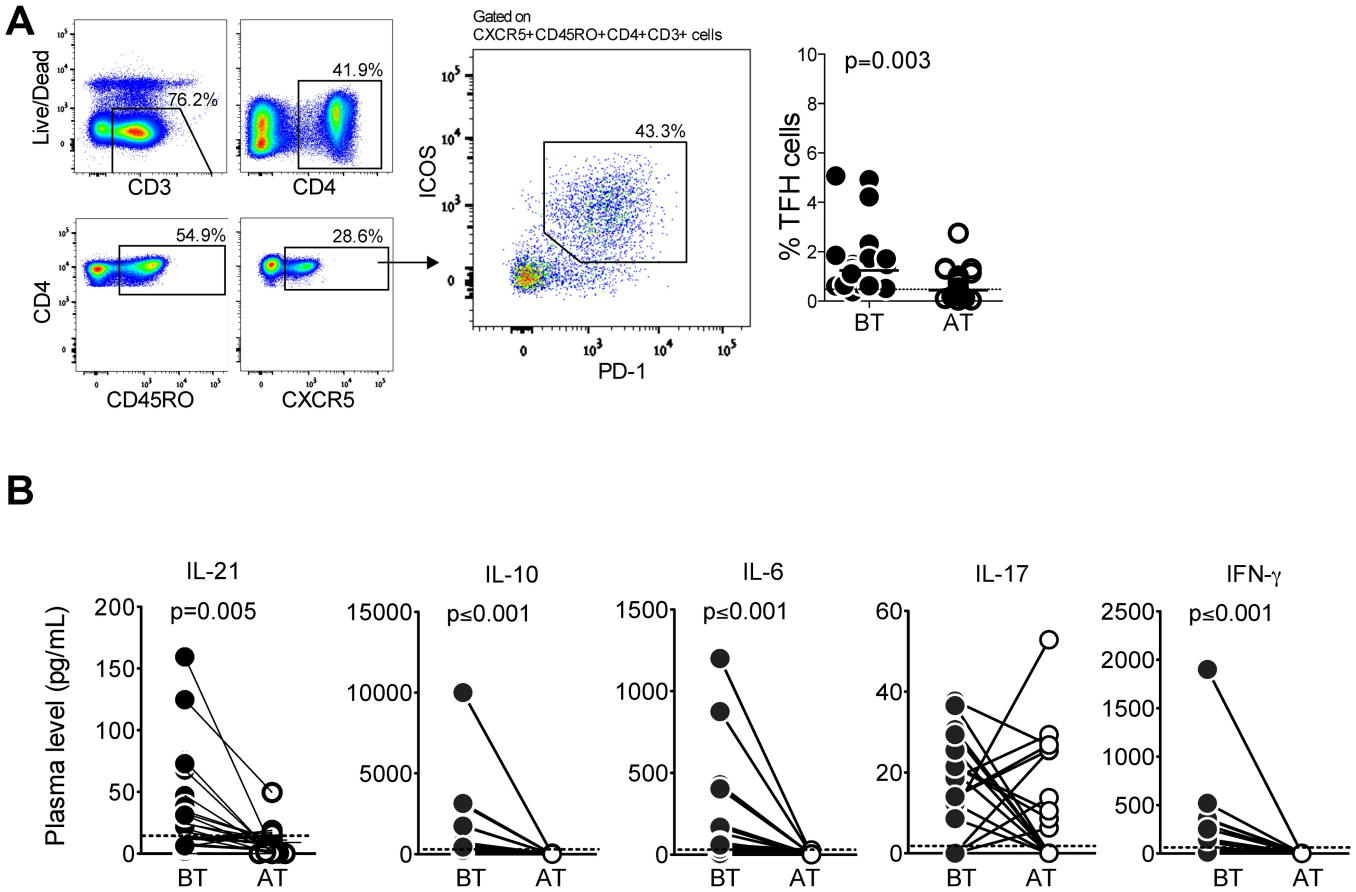
*Plasmodium vivax*-infected patients display higher frequency of circulating Tfh cells and increased levels of Tfh cells like cytokines. PBMC and plasma from *P*. *vivax*-infected patients were analyzed ex vivo (n = 24) during acute malaria episode (BT) and 30–45 days after treatment (AT). **A**. Gating strategy for the analysis of Tfh cells based on the simultaneous expression of PD-1^+^ICOS^+^CXCR5^+^CD45RO^+^CD4^+^CD3^+^ within live PBMC (density plots, counter clockwise) and scattered plots (right panel) showing the distribution of the percentage of Tfh cells in *P*. *vivax*-infected patients before (BT) and after treatment (AT). **B**. IL-21, IL-6, IL-10, IL-17 and IFN-*γ* were measured in plasma of *P*. *vivax*-infected patients before (filled circles) and after (open circles) treatment. Lines represent median values of the given measurement in each group. Dotted lines represent healthy donors. *p* values are depicted in the figure.

To characterize Tfh cells, we examined the simultaneous expression of CD3, CD4, CD45RO, CXCR5, ICOS and PD-1 by CD4^+^ T cells among total PMBC (PD-1^+^ICOS^+^CXCR5^+^CD45RO^+^CD4^+^CD3^+^ cells), and, consistent with the data described above, we observed a significant higher frequency of circulating Tfh cells during acute malaria compared to the same patients AT (Fig 2A) and to HD (S3 Table). Moreover, significantly higher levels of IL-21 were observed in plasma from *P*. *vivax*-infected patients BT compared to AT. Higher levels of IL-6, IL-10 and interferon-gamma (IFN-γ) were also found during acute malaria (Fig 2B). These observations suggest that *P*. *vivax* infection triggers production of IL-21 and promotes Tfh cells expansion.

### Tfh cells from *P*. *vivax*-infected patients secrete IL-21 and induce immunoglobulin production by naïve B cells

A hallmark of Tfh cells is production of IL-21, which drives the growth and differentiation of B cells and isotype switching [21]. To evaluate whether Tfh cells from *P*. *vivax*-infected patients secreted IL-21 and contributed to B cells differentiation, distinct CD4^+^ T cell subsets were FACS-sorted and cultured with different B cell subsets (S1A Fig). We observed that Tfh cells from *P*. *vivax*-infected patients produced significantly higher levels of IL-21 than memory or naïve CD4^+^ T cells when co-cultured with different B cell subsets in the presence or absence of staphylococcal endotoxin B (SEB) (S1B Fig). In addition, in the presence of *P*. *vivax*-infected reticulocytes or SEB, we observed increased IgG production by naïve B cells co-cultured with Tfh cells compared to naïve B cells co-cultured with memory CD4^+^ T cells (S1C Fig). Moreover, higher frequency of IL-21 producing Tfh cells was observed among acutely malaria patients when compared to HD (S5 Fig). Taken together, these findings suggest that IL-21 producing Tfh cells play an important role in the activation of B cells and antibody production during malaria.

### *P*. *vivax* induces the expansion of CXCR3^+^CCR6^-^ and CXCR3^-^CCR6^+^ subsets of Tfh cells

**Fig 3:**
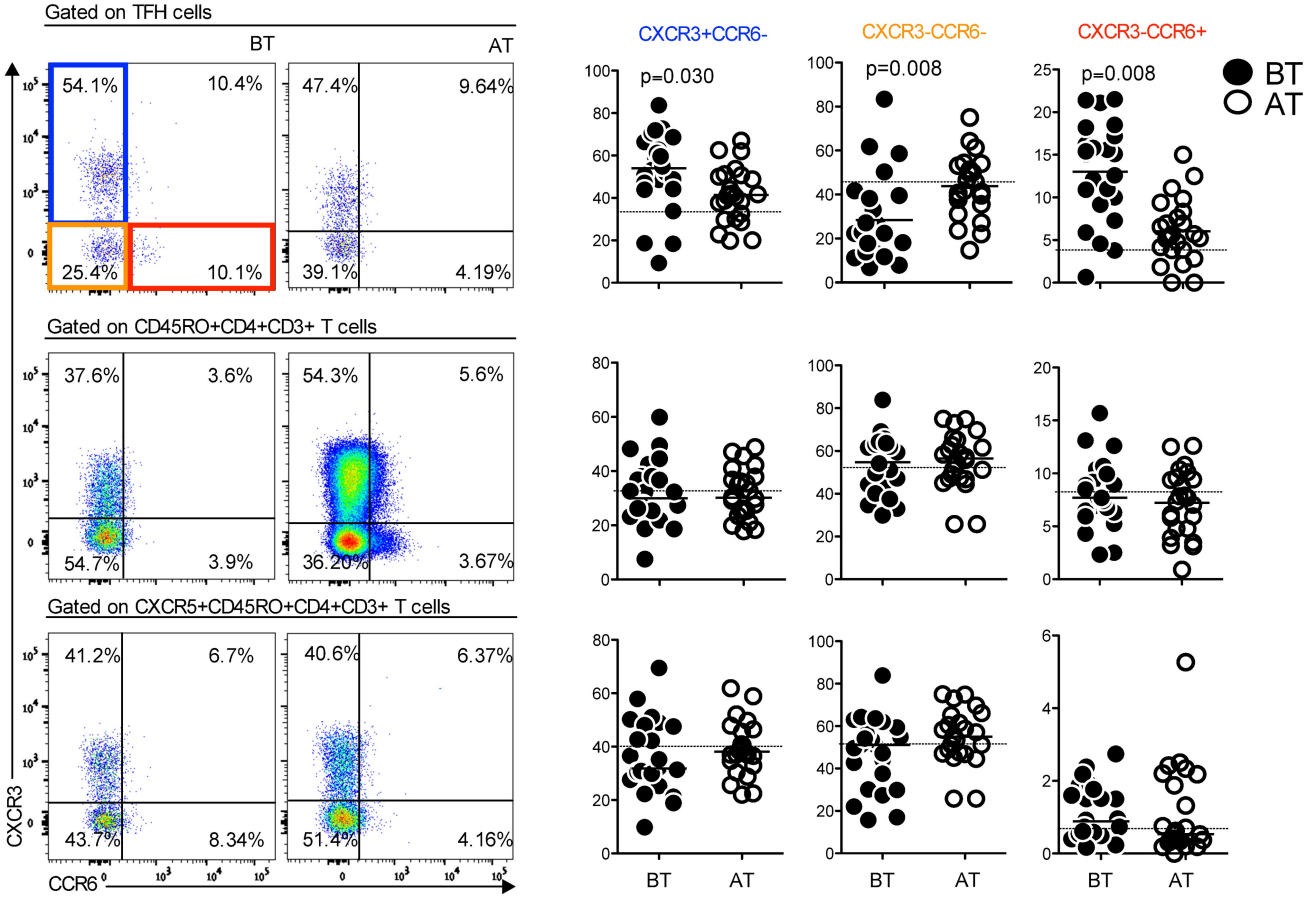
Distribution of CXCR3 and CCR6 expressing cells is altered in the Tfh cells compartment during *Plasmodium vivax* infection. PBMC from *P*. *vivax*-infected patients were analyzed ex vivo (n = 24) during acute malaria episode (BT) and 30–45 days after treatment (AT). All the T cell subsets were analyzed after gating on live CD3^+^ cells. Representative density plots showing frequency of Tfh cells (PD-1^+^ICOS^+^CXCR5^+^CD45RO^+^CD4^+^CD3^+^ cells), CD45RO^+^CD4^+^ and CXCR5^+^CD45RO^+^CD4^+^ T cells expressing or not CXCR3 and CCR6 from a single *P*. *vivax*-infected patient. Scattered plots showing the percentage of T cell subsets in *P*. *vivax*-infected patients before (BT) and after treatment (AT) expressing or not CXCR3 and CCR6. Lines represent median values of the given measurement in each group. Dotted lines represent healthy donors. *p* values are depicted in the figure.

Characteristics of Tfh cells may partially overlap with Th1, Th2, and Th17 cells, contributing to the plasticity of the Tfh cells lineage [11]. To determine whether such flexibility was altered during malaria we evaluated the expression of CXCR3 and CC chemokine receptor 6 (CCR6) by CD4^+^ T cell populations in *P*. *vivax*-infected patients BT and AT. We found that *P*. *vivax* infection did not alter the proportions of CXCR3^+^CCR6^-^ (Th1), CXCR3^-^CCR6^-^ (Th2) and CXCR3^-^CCR6^+^ (Th17) cells within the memory (CD45RO^+^) and activated memory (CXCR5^+^ CD45RO^+^) CD4^+^ T cell subsets (Fig 3, middle and lower panels). However, the frequencies of CXCR3^+^CCR6^-^ and CXCR3^-^CCR6^+^ Tfh cells were significantly higher during acute malaria when compared to the same patients AT (Fig 3, upper panels). In addition, a significantly lower frequency of CXCR3^-^CCR6^-^ Tfh cells was found BT (Fig 3, upper panels). These data suggest that *P*. *vivax* infection selectively influences the plasticity of Tfh cells.

### Levels of *P*. *vivax*-specific IgG subclasses are increased during acute malaria and decrease shortly after treatment

**Fig 4:**
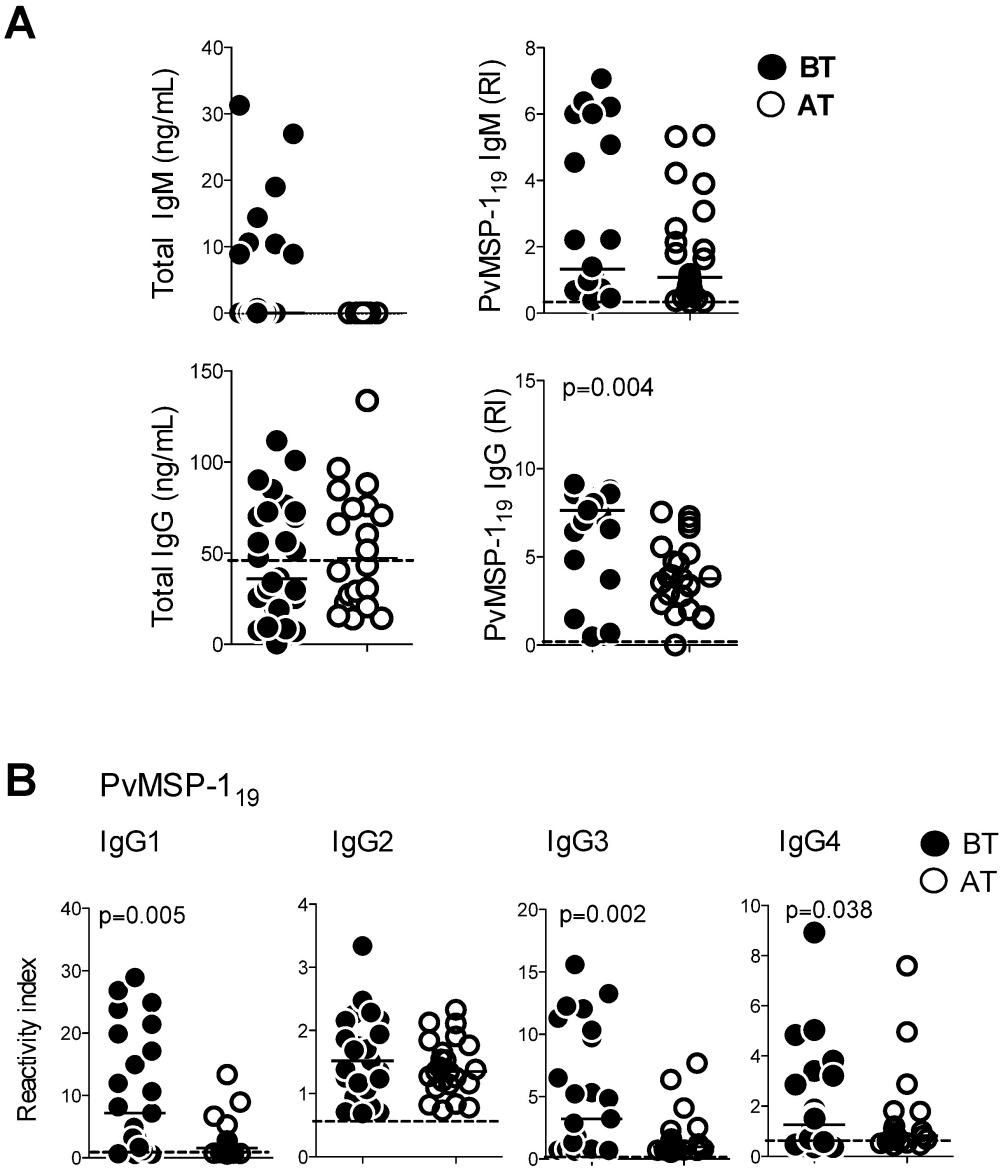
Increase in the reactivity index of IgM and IgG against MSP-1_19_ from *Plasmodium vivax* during malaria. **A.** Total and *Plasmodium vivax*-specific antibodies were measured by enzyme-linked immunosorbent assay (ELISA). Total and PvMSP-1_19_ IgM and IgG were measured in plasma of patients during acute malaria (BT) and after treatment (AT). **B.** IgG subclasses against PvMSP-1_19_ were measure in plasma of patients during acute malaria (BT) and after treatment (AT). Lines represent median values of the given measurement in each group. Dotted lines represent healthy donors. *p* values are depicted in the figure.

We next assessed circulating levels of total or 19-kDa Merozoite Surface Protein-1 (PvMSP-1_19_)-specific IgM and IgG in *P*. *vivax*-infected patients, BT and AT. As expected, total IgM and IgG levels were not altered upon infection (Fig 4A, left panels). Furthermore, although acutely infected patients displayed similar levels of MSP-1_19_-specific IgM, they had higher levels of MSP-1_19_-specific IgG when compared to the same patients AT (Fig 4A, right panels). It has been described that IL-21 is a switch factor for the production of IgG1 and IgG3 by human B cells [22]. Indeed, we observed that the reactivity indices of anti-PvMSP1-_19_ IgG1, IgG3 and IgG4 antibodies were significantly higher during malaria when compared to the same patients AT (Fig 4B). Antibody levels against AMA-1 were assessed in the same patients (S6 Fig). Distinct from MSP-1_19_-specific immunoglobulin levels, acutely infected patients had higher levels of AMA-1-specific IgM but similar levels of IgG when compared to the same patients AT (S6A Fig). Moreover, different from MSP-1_19_-specific antibodies, the levels of circulating IgG2 and IgG4 were higher after treatment than before treatment (S6B Fig). These results indicate that different *P*. *vivax* antigens may trigger distinct malaria specific IgM and IgG responses and that MSP-1_19_-specific antibodies are induce during acute infection and their levels quickly decrease after treatment.

### *P*. *vivax* infection alters the memory B cell compartment and triggers expansion of plasma cells

**Fig 5:**
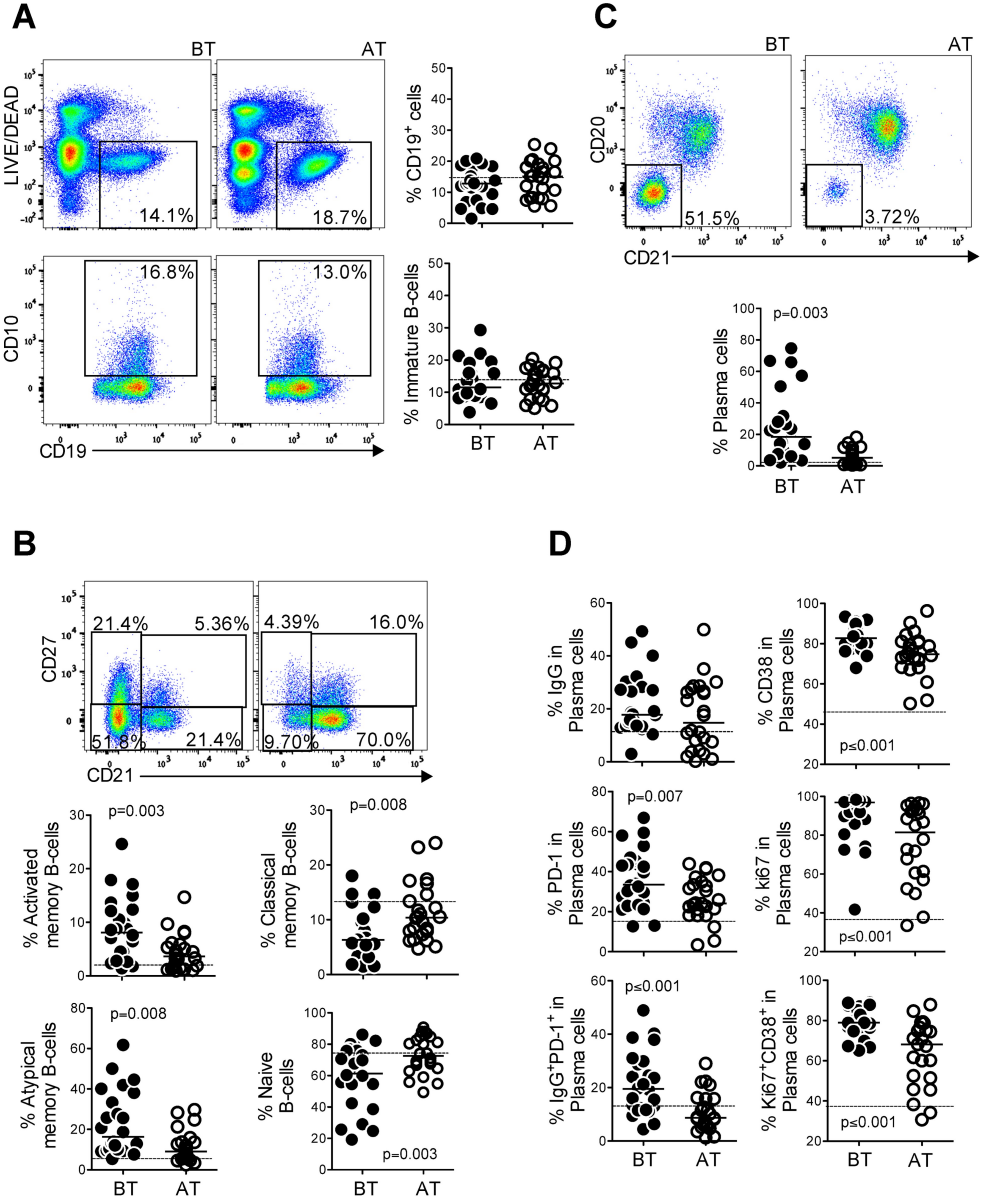
Proportions of B cell subsets are altered during *Plasmodium vivax* infection. PBMC from *P*. *vivax*-infected patients were analyzed ex vivo (n = 24) during acute malaria episode (BT) and 30–45 days after treatment (AT). All the B cell subsets were analyzed after gating on live CD19^+^ cells. **A**. Representative density plots showing frequency of total B cells (CD19^+^ cells), immature (CD10^+^) B cells from a single *P*. *vivax*-infected patient and scattered plots showing the proportions of total B cells (CD19^+^ cells) and immature (CD10^+^) B cells. **B.** Representative density plots showing frequency of activated memory (CD27^+^CD21^-^), classical memory (CD27^+^CD21^+^), atypical memory (CD27^-^CD21^-^) and naïve (CD27^-^CD21^+^) B cells and scattered plots showing the distribution of the B cell subsets described above in *P*. *vivax*-infected patients. **C.** Representative density plots showing the proportion of plasma cells (CD21^-^CD20^-^) from a single *P*. *vivax*-infected patient before and after treatment and scattered plots showing their distribution. **D.** Frequency of IgG, CD38, Ki67, PD-1, IgG and PD-1 and Ki67 and CD38 expressing plasma cells. Lines represent median values of the given measurement in each group. Dotted lines represent healthy donors. *p* values are depicted in the figure.

Since IL-21 levels and IgG responses were elevated during malaria, we sought to investigate whether *P*. *vivax* infection altered the proportions of circulating B cell subsets. We found that *P*. *vivax* infection did not change the frequency of either total B cells or immature B cells (Fig 5A). However, when B cell subsets were assessed, we observed the proportions of activated memory (CD27^+^CD21^-^) and atypical memory (CD27^-^CD21^-^) B cells were significantly higher in *P*. *vivax* infected patients than in the same individuals AT. Conversely, treatment was associated with significantly higher frequencies of classical memory (CD27^+^CD21^+^) and naïve (CD27^-^CD21^+^) B cells (Fig 5B).

Since immunity to malaria requires repeated exposure to parasites and can be lost in absence of infection, we investigated the proportion of plasma cells during infection and AT (Fig 5C). Plasma cells have been defined as B cells that express CD38 but not CD20 [23], or as B cells that do not express both CD20 and CD21 [24,25]. Since most CD20^-^CD21^-^ B cells are also CD38^+^, the majority of cells identified using these strategies are coincident. We found that *P*. *vivax* infection triggered a significant increase in the frequency of plasma cells (CD21^-^CD20^-^ within CD19^+^ B cells), which decreased AT to levels similar to those found in HD (Fig 5C, S3 Table). However, there were no significant changes in the percentage of IgG-expressing plasma cells in the circulation, either BT or AT (Fig 5D). Nevertheless, *P*. *vivax* infection induced increased frequencies of PD-1^+^, CD38^+^ and Ki67^+^ plasma cells, as well as subsets co-expressing IgG/PD-1 and Ki67/CD38, indicating activation (Fig 5D). Although the proportions of CD38^+^, Ki67^+^ and CD38^+^Ki67^+^ plasma cells were diminished AT, they remained above the levels observed in HD (Fig 5D, S3 Table). Together, these observations indicate that treatment triggered a contraction in plasma cells but was not sufficient to decrease their activation status to physiological levels.

### Patients repeatedly infected by *P*. *vivax* have higher levels of parasite specific IgG and increased proportions of Tfh cells

**Fig 6:**
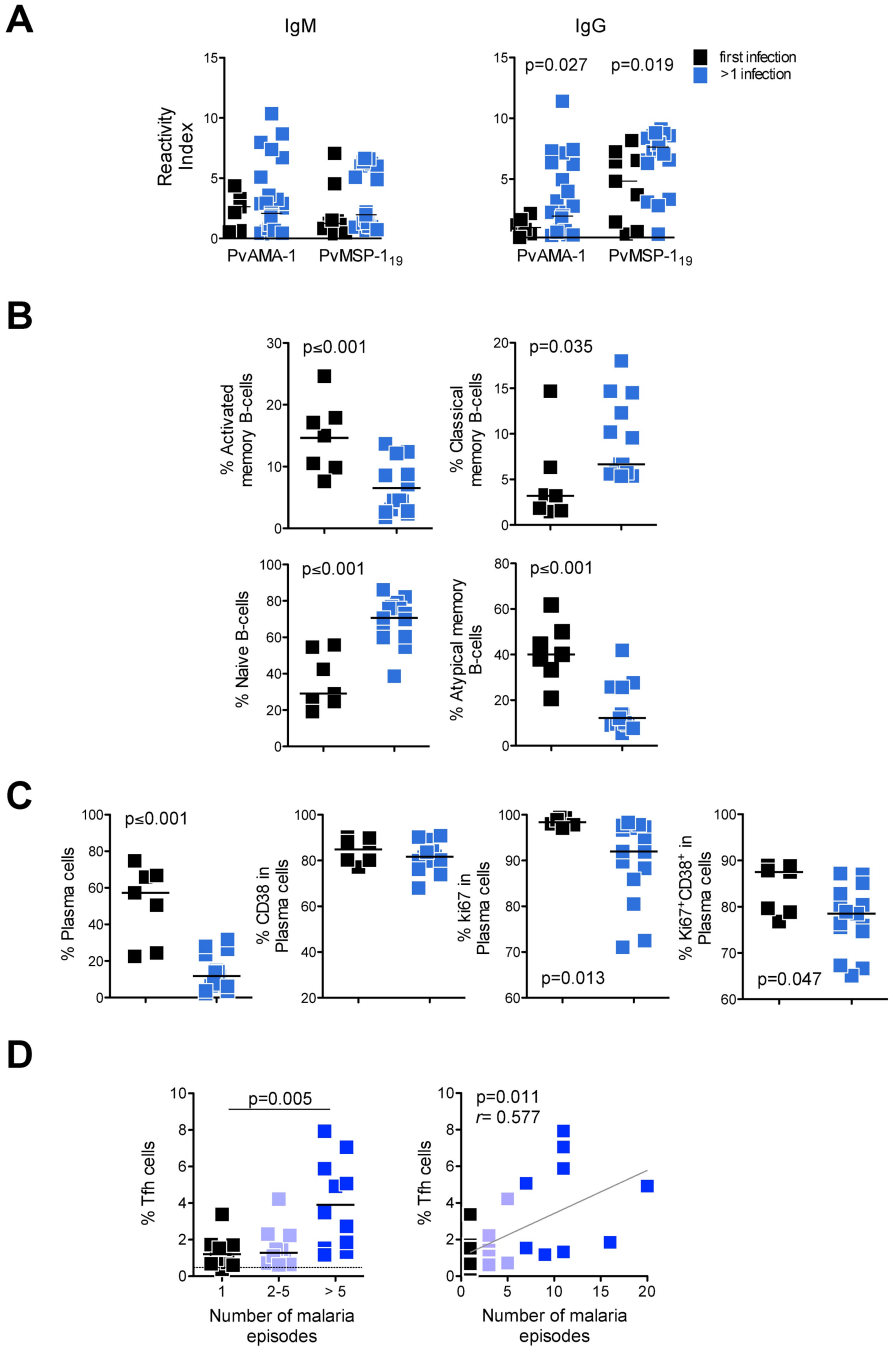
Repeated *Plasmodium vivax* exposures increase the levels of antigen-specific IgG. **A-C.** Components of humoral response were measured in patients acutely infected for the first time (black squares) or with multiple infections (blue squares) with *P*. *vivax*. **A.** Pv AMA-1 and PvMSP-1_19_ IgM and IgG were measure in plasma. **B.** Scattered plots showing frequency of activated memory (CD27^+^CD21^-^), classical memory (CD27^+^CD21^+^), naïve (CD27^-^CD21^+^) and atypical memory (CD27^-^CD21^-^) B cells in *P*. *vivax*-infected patients described above. **C.** Scattered plots showing the proportion of plasma cells (CD21^-^CD20^-^) and IgG, CD38, Ki67, PD-1, IgG and PD-1 and Ki67 and CD38 expressing plasma cells from *P*. *vivax*-infected patients. All the B cell subsets were analyzed after gating on live CD19^+^ cells. **D.** Frequency of Tfh cells (PD-1^+^ICOS^+^CXCR5^+^CD45RO^+^CD4^+^CD3^+^) cells are shown in patients infected for the first time (black squares) or infected 2 to 5 times (grey squares) or more than 5 times (blue squares) with *P*. *vivax*. Lines represent median values of the given measurement in each group (left graph). Correlation between number of malaria episodes and proportion of Tfh cells (right graph). *p* values are depicted in the figure.

When patients were stratified based on the number of malaria infections, higher levels of IgG antibodies specific for Apical Membrane Antigen 1 (PvAMA-1) and PvMSP-1_19_ were observed in individuals who were repeatedly infected with the parasite (Fig 6A). Moreover, this stratification revealed that multiple infections modulated the proportions of B cell subsets (Fig 6B–6D, S4 Table). While patients with acute malaria displayed an increase in the proportion of activated memory and atypical memory B cells compared to HD or patients AT (Fig 5B, S3 Table), this was not the case in patients who had previously been infected by *P*. *vivax* since they had lower frequencies of these two subsets (Fig 6B). In addition, patients undergoing primary infection displayed significantly higher frequencies of total plasma cells and of Ki67^+^ and Ki67^+^CD38^+^ expressing plasma cells compared to those who had experienced prior infection (Fig 6C). Conversely, significantly higher proportions of classical memory B cells were found in patients who had been infected on multiple occasions (Fig 6B). Moreover, patients who experienced more than five malaria episodes had increased proportions of circulating Tfh cells, compared to patients undergoing primary infection (Fig 6D, left graph). A significant positive correlation analysis was observed when numbers of malaria episodes were plotted against proportions of Tfh cells (Fig 6D, right graph). When the same analyses were performed after treatment, the differences between patients experiencing their first infection versus those who had more than one malaria episode were lost, suggesting that the presence of the parasite boosts the B cell response and determines its pattern (S2 Fig).

We then performed a detailed correlation analysis between the various cell types analyzed and the soluble parameters assessed. The analysis revealed that before treatment, malaria patients, regardless of the number of infections, presented three clusters of nodes with large number of neighborhood connections: (i) B-cell subsets, (ii) plasma cell subsets and (iii) anti-MSP1/anti-AMA antibodies (S3 Fig). When patients were categorized according to the number of malaria episodes, these clusters were segregated into distinct subgroups of patients showing relevant connections with Tfh cells. Thus, patients undergoing primary infection presented a cluster of nodes with connections preferentially composed by B-cell subsets, particularly classical and atypical B-cells. Patients who experienced 2–5 malaria episodes exhibited most connections among antibodies, while patients who had more than 5 malaria episodes displayed a less intricate network, but strong correlations involved the plasma cell cluster, antibodies and Tfh cells.

**Fig 7:**
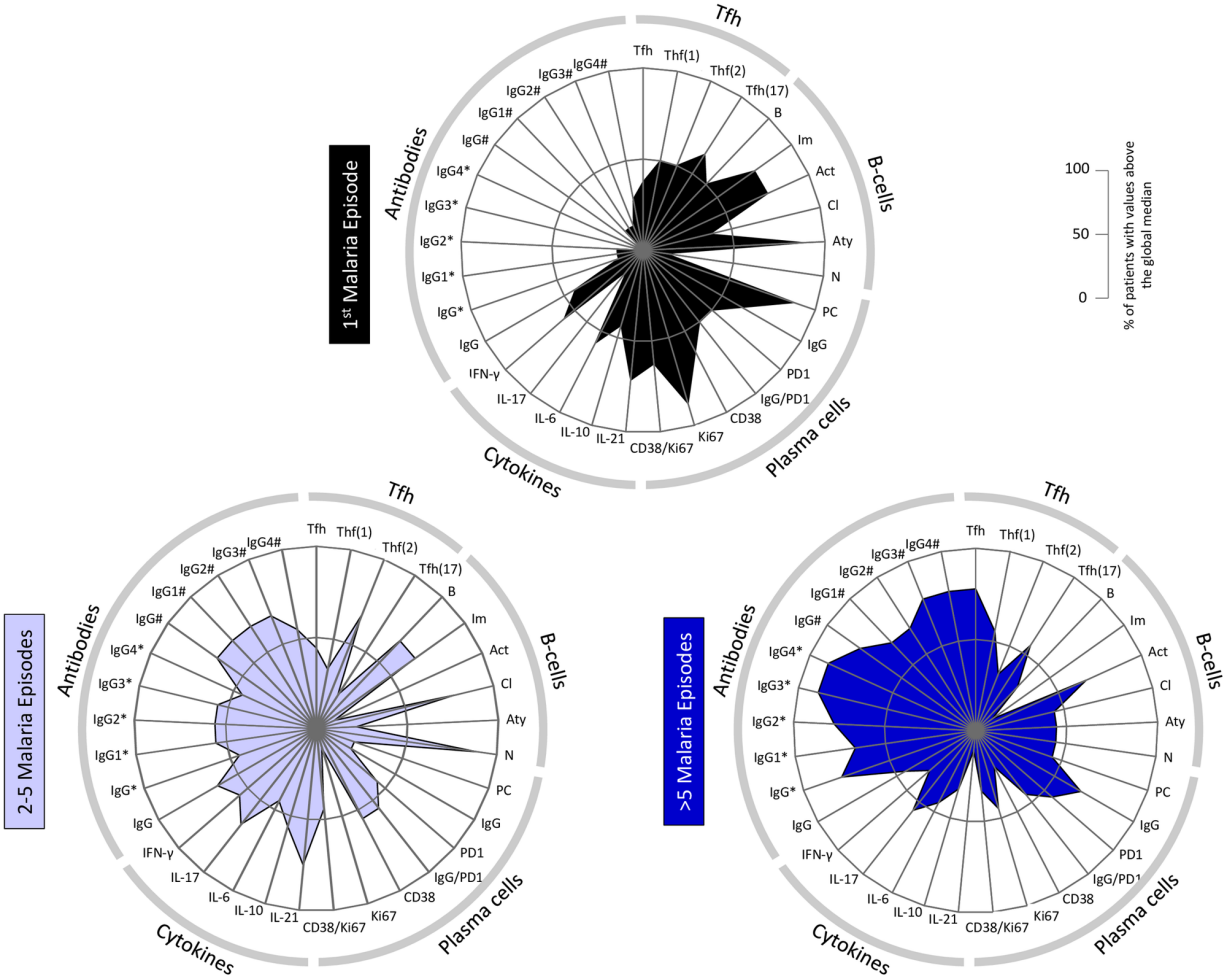
Repeated *Plasmodium vivax* exposures alter the distribution of the high produces of immunological markers. Radar graphs represent the high producers of Tfh cells, B cells, plasma cells and their subsets, and the levels of cytokines and antibodies (clockwise) assessed in patients undergoing the first malaria (upper panel) or who had between 2–5 lower, left panel) or more than 5 malaria episodes (lower, right panel). The inner circle represents the 50^th^ percentile for each parameter, which was taken as threshold to define relevant frequency of patients with higher levels of a given biomarker. * and # represent, respectively, immunoglobulins specific for AMA-1 and MSP-1_19_.

Moreover, we plotted in radar graphs all the parameters evaluated considering the number of malaria episodes. Tfh cells, B cells, plasma cells and their subsets, cytokines and antibodies are represented clockwise (Fig 7). The inner circle represents the 50^th^ percentile, which was taken as threshold to segregate higher and lower expression/production. The immunological parameters evaluated in patients undergoing the first malaria fill a small area of the radar graph, especially the area representing the antibodies (Fig 7, upper panel). Some of the cell subsets, such as immature, activated, atypical B cells and plasma cells are expanded in this group of patients. There is a clear expansion of the area composed by the antibodies subclasses as the patients are repeated exposed by *P*. *vivax* (Fig 7, lower panels). The main alterations observed in the B cell compartment are the increase of the area occupied by classical memory and decrease of the area occupied by atypical memory B cells in patients who had from 2 to 5 malaria episodes (Fig 7, lower, left panel). As the number of malaria episodes increases, the areas representing antibody levels and proportions of Tfh cells increase (Fig 7, lower, right panel).

These data indicate that re-infection by *Plasmodium vivax* triggers circulating Tfh cells, promotes differentiation of B cells into classical memory B cells and boosts antibody levels that together provide an efficient humoral immune response.

## Discussion

While the role of Tfh cells in the GC reaction has been reported in several studies, the presence and function of their circulating counterpart has only been recently accepted. Our findings clearly demonstrate that *P*. *vivax* infection triggers an increase in the proportion of circulating Tfh cells that are the main source of IL-21 and are able to induce immunoglobulin production by naïve B cells. Our observations are consistent with reports demonstrating that CXCR5^+^CD4^+^ T cells represent memory Tfh cells that regulate B cell responses [11].

Memory cells are long-lived, and their longevity is dependent on their ability to undergo homeostatic proliferation in the absence of antigen [26]. *P*. *vivax* infection does not alter the frequencies of memory and CXCR5 expressing CD4^+^ T cell compartments. However, the infection induced expression of activation and co-stimulatory molecules, ICOS and CD40L, on CD4^+^ T cells, known to be crucial for T-B interactions providing help for B cell activation, maturation and antibody production [19,27,28,29]. Among the T lymphocytes, Tfh cells are the specialized subset in helping B cell responses [30].

A rigorous characterization of GC Tfh cells in both mice and humans takes into account the expression of CD3, CD4, CD45RO, CXCR5, ICOS, PD-1 and the transcription factor Bcl-6 [10,31,32]. It has been reported in mice that, after providing help to B cells, Tfh cells may exit the GC, downregulate Bcl-6, and circulate in the blood [33]. Based on this phenotype, a significantly higher frequency of circulating Tfh cells is found during malaria infection in humans. It is well accepted that immunoglobulin production is essential to an effective immune response against *Plasmodium* and this production is T-dependent [34]. However, in human, there is only one published report in malaria caused by *P*. *falciparum*, demonstrating that circulating PD-1^+^CXCR5^+^CD4^+^ T cells from patients have characteristics of GC Tfh cells, and our study represents the first report of an increase in Tfh cells following *P*. *vivax* infection [19,35].

Consistent with the expansion of Tfh cells during *P*. *vivax* infection, we found increased levels of IL-21, a key cytokine produced by Tfh cells that, along with IL-4 and IL-10, promotes growth, differentiation and class switching of B cells [36]. A better understanding of the ability of the circulating Tfh cells to promote B cell differentiation into plasma cells is still lacking. By purifying T and B cell subsets we demonstrated that circulating Tfh cells from malaria infected patients are the main source of IL-21 and trigger antibody production by naïve B cells. Higher levels of IL-10, IL-6 and IFN-*γ* were also observed in the circulation of *P*. *vivax*-infected patients. Cytokines play essential roles in all phases of Tfh cells differentiation and several studies show that infections regulate the development and activity of this cell subset through modulation of cytokine production. lL-6 participates in the induction of early differentiation of Tfh cells, mainly acting through either Signal Transducer and Activator of Transcription 1 (STAT1) or STAT3 to trans-activate Bcl-6 [37]. Large amounts of IL-10 are also detected in cultures of CXCR5^+^CD4^+^ T and naive B cells, and blocking IL-10 results in a partial inhibition of immunoglobulin production [11]. On the other hand, excessive IFN-*γ* and TNF may limit Tfh cells function and GC B cell responses during the blood stage of experimental *Plasmodium* infection [38,39]. Another very recent study showed that experimental *Plasmodium* infection-induced type I IFN limit Tfh cells accumulation and humoral immunity though secretion of IL-10 and IFN-γ by T regulatory cells [40]. Moreover, it was described that IFNAR1-signalling is associated with impaired GC B cell formation, antibody production and Tfh cell differentiation [41].

Studies suggest that the Tfh cells compartment is heterogeneous and that some Tfh cells are able to secrete cytokines characteristic of other T helper cell subsets [34,42]. Depending upon stimulus and microenvironment, Tfh cells can express T-box transcription factor (Tbet), transcription factor GATA-binding protein 3 (Gata3), or retinoic acid-related orphan receptor-gamma T (RORγt), which results in a diversity of Tfh cell subsets producing low levels of other Th-like cytokines with different abilities to regulate B cell responses [34,43]. Tfh cell subsets can also be distinguished based on the expression of the chemokine receptors CXCR3 and CCR6: CXCR3^+^CCR6^−^ cells expressed T-bet, CXCR3^−^CCR6^−^ cells expressed GATA3 and CXCR3^−^CCR6^+^ cells expressed RORγT [11,44]. *P*. *vivax* infection induced an increase in both CXCR3^+^CCR6^-^ and CXCR3^-^CCR6^+^ Tfh cell subsets and a lower frequency of CXCR3^-^CCR6^-^ expressing cells. These alterations were specific to Tfh cells, since no differences were observed in memory and activated memory CD4^+^ T cells. According to Obeng-Adjei and colleagues (2015), during malaria caused by *P*. *falciparum*, the CXCR3^-^ Tfh cell subset is better than the Th1-like CXCR3^+^ subset in helping B cells, but no correlation was found between Tfh cells and B lymphocytes or immunoglobulin production [19].

Antibodies are crucial to naturally acquired protective immunity against blood stage malaria, with functions that include inhibition of merozoite invasion, blocking cytoadherence, and improving phagocytic activity of monocytes and macrophages [45]. In this work, infected patients displayed higher levels of MSP-1_19_-specific IgG than the same patients AT. The same was not observed for AMA-1-specific total IgG response. Moreover, a more integrated analysis of the proportion of the patients considered to represent the high producers of antibodies show that the more malaria episodes the patient had, the more antigen-specific antibodies they produce. Indeed, a meta-analysis study discuss that despite a great heterogeneity of humoral response observed where *P*. *vivax* infection is endemic, IgG response to a number of antigens is associated with increase antibody levels [46]. The same is observed when the dynamics of the antibody response is assessed during *P*. *falciparum* infection. Despite the antibody response seems to be short-lived, data revealed a modest increase in antibody reactivity with age [47]. The same group showed that the expansion of memory B cells and antibody compartments depends on parasite exposure rather than age [48]. Increased reactivity indices of IgG1 and IgG3 anti- PvMSP-1_19_ were observed during acute malaria [49]. PvMSP-1_19_-reactive-IgG1 antibodies predominate in individuals living in Brazilian endemic areas with different levels of exposure. The association between isotypes and protection to *P*. *falciparum* infection is not clear but it has been accepted that IgG1 and IgG3 are considered cytophilic and protective, whereas IgG2 and IgG4 may even block protective mechanisms [50]. Higher levels of IgG1 and IgG3 are also observed in subjects with long-term exposure in *P*. *vivax* infection [51].

Previous data reported a decrease in total B cells following acute *P*. *falciparum* and *P*. *vivax* infections [52]. Moreover, another study show that some exposure to P. falciparum does not result in stable populations of antigen-specific memory B cells [53]. More recently, the decrease in B lymphocytes was associated with an expansion of transitional (immature) B cells in children following *P*. *falciparum* infection [54]. Our study shows that *P*. *vivax* infection does not alter proportions of total and immature B cells, but striking changes are seen in memory and naïve compartments. It is believed that recurrent infections are necessary to maintain acquired immunity to malaria and avoid short-lived antibody responses due to defective or suboptimal responses of memory B cells [5]. Indeed, individuals living in endemic areas develop high levels of *Plasmodium*-specific antibodies and exhibit resistance to malaria infections, or at least to clinical symptoms [55]. However, it has been described that repeated malaria infections in areas of high endemic exposure can lead to B cell anergy or exhaustion and expansion of atypical memory B cells [25]. The role of atypical memory B cells in the context of malaria remains unclear. Atypical memory B cells represent up to 40% of all circulating B lymphocytes [25], but they are uncommon in healthy individuals living in malaria-free regions. It has been postulated that they contribute to the production of short-lived antibodies due to the generation of short-lived plasma cells [56]. However, others have raised the possibility that their expansion during *P*. *falciparum* infection is beneficial and may promote protection from clinical disease by modulating the immune response [25]. Our results show that *P*. *vivax* infection triggers higher proportions of atypical memory B cells and lower frequencies of classical memory B cells. Previous study showed that higher proportions of IgD^-^ atypical memory B cells are observed in *P*. *vivax* exposed pregnant and non-pregnant women compared to non-exposed individuals [57]. An controlled *P*. *vivax* human infection study showed an enhanced ability of atypical memory B cells to proliferate just after treatment initiation, which was lost 35 days later [58]. Atypical memory B cells were increased in patients living in low transmission *P*. *falciparum* area, and further increase in high transmission region [24]. Interestingly, even in our study area where malaria is not highly endemic, single- and multiple-infected individuals displayed distinct proportions of atypical and classical memory B cells. Although atypical memory B cells were increased during acute malaria, first time infected patients had higher frequencies compared to individuals who had more than one episode of malaria. The contrary was observed for classical memory B cells; individuals who had more than one malaria episode had higher proportions of this cell subpopulation. The alterations observed in atypical and classical cell subsets, promoting the decrease of the latter and the increase of the former is accompanied by an important increase in the antigen-specific antibody levels. The changes observed in the balance between atypical and classical memory B cells in patients with multiple infections suggests that an efficient memory response following repetitive malaria exposure is maintained by classical memory B cells.

*P*. *vivax* infection also induces expansion of plasma cells and their activation as assessed by IgG, PD-1, CD38 and Ki67 expression. However, when patients were segregated by malaria episodes, higher proportions of plasma cells and Ki67^+^ and Ki67^+^CD38^+^ plasma cells were found in first-time infected individuals. Despite this observation, it is important to mention that even with lower proportions of plasma cells, multiply-infected patients produced higher levels of *P*. *vivax*-specific IgG. A possibility is that plasma cells from re-exposed patients migrate from the circulation to other lymphoid tissues, such as bone marrow, or even that they respond more efficiently than their counterparts from individuals undergoing primary infection. Indeed, in a highly malaria transmission endemic area, very short-lived antibody responses to malaria were associated with younger individuals who had the fewest number of malaria infections [56], suggesting that long-lived humoral responses develop after repeated infections.

In summary, *Plasmodium vivax* infection triggers an increase in the proportion of circulating Tfh cells. Purification of Tfh cells from malaria patients confirmed that these were the main source of IL-21 and therefore likely to play an important role in the induction of protective humoral immunity. *P*. *vivax* infection also induces changes in B cell compartments, with multiple infections driving an increase in classical memory B cells that was accompanied with high levels of specific antibodies. The identification of *bona fide* circulating Tfh cells during *P*. *vivax* infection suggests that novel vaccination strategies should aim to prime strong Tfh cells responses in order to generate effective and long-lasting humoral immunity.

## Methods

### Patients and healthy donors

*P*. *vivax*-infected patients (n = 24, 87.5% male and 12.5% female, 18–56 years old) with uncomplicated malaria were included in this study and received medical care at Centro de Pesquisa de Medicina Tropical de Rondônia in Porto Velho, Rondônia, a malaria endemic area in Brazil. Peripheral blood was collected from adults, 18 years or older individuals, BT and after being diagnosed with *Plasmodium vivax* infection by thick blood smear film. Blood samples were collected again 30–45 days AT. Infection by a single *Plasmodium* species was confirmed by polymerase chain reaction (PCR). Patients were treated according to the Brazilian Ministry of Health guidelines. Clinical characteristics and laboratory data are shown in S1 Table. Blood samples were also obtained from healthy donors (HD), who never had malaria, from Porto Velho (2 female and 4 male, 18–50 years old).

### Ethics statement

This study was performed under protocols reviewed and approved by the Ethical Committees on Human Experimentation from Centro de Pesquisas René Rachou, Fiocruz (CEP-CPqRR 665281, CAAE: 30492014.9.0000.5091). All patients were adults and were enrolled in the study after providing written informed consent.

### Immunoglobulin levels

Total levels of IgM and IgG were measured in culture supernatants using Human IgG and IgM total enzyme-linked immunosorbent assay (ELISA) Ready-Set-Go! (eBioscience) according to the manufacturer’s instructions. IgM, IgG and IgG’s subclasses anti-AMA-1 and anti-MSP-1_19_ were measured in plasma and supernatants of cell cultures by ELISA. ELISA plates were coated with the recombinant proteins PvAMA-1 and PvMSP-1_19_ (1μg/well) produced as previously described [59,60]. Plasma samples were added to each well at a final dilution of 1:100. The presence of bound IgA, IgM, IgG and subclasses of IgG was detected using tetramethylbenzidine (Sigma) at 10mg/mL diluted in phosphate-citrate buffer (pH 5.0) containing hydrogen peroxide (0.03% [vol/vol]). The final optical density (OD) at 450nm was obtained by using a VERSAmax microplate reader. The results were expressed as reactivity index (RI = the ratio between the OD 450nm values obtained from the sample and the values of the cut-off). Cut-off points were set at three standard deviations above the mean OD 450nm of plasma from 24 individuals who had never been exposed to malaria. Values of RI > 1.0 were considered positive [27].

### Immunophenotyping and intracellular cytokine assessment

PBMC from heparinized peripheral blood samples were prepared by centrifugation with Ficoll-Hypaque gradient (GE Healthcare Life Sciences) and then frozen in fetal bovine serum (FBS) (GIBCO, Life Technologies) with 20% dimethyl sulphoxide (SIGMA). PBMC were thawed in RPMI 1640 (Sigma-Aldrich) 10% FBS and 20U/mL benzonase nuclease (Novagen). Cells were washed in phosphate-buffered saline (PBS), and stained with Violet or Acqua Live/Dead (Invitrogen) and with monoclonal antibodies. PBMC were washed, fixed and permeabilized (FoxP3 staining buffer Set, eBioscience) according to the manufacturer’s instructions. Cells were incubated with antibodies against intracellular proteins, washed, fixed and acquired on an LSR-FORTESSA. The antibodies used to define Tfh cells and B cell subpopulations are described in S2 Table.

### Analyses of T and B cell subpopulations

Doublets were removed using a forward scatter area (FSC-A) *versus* height (FSC-H) gate. Events were gated in function of Time *versus* size scatter area (SSC-A) and several combinations of fluorochromes to exclude debris flux interruptions. Dead cells were excluded using a Live/Dead gate *versus* CD3 or CD19 to phenotype T and B cells respectively (Fig 1). The following combinations of molecules were used to define specific cell subsets: T cells: CD4+CD45RO- naïve cells; CD4^+^CD45RO^+^CXCR5^-^ memory cells; CD4^+^CD45RO^+^CXCR5^+^ activated memory cells; CCD4^+^CD45RO^+^CXCR5^+^ICOS^+^PD-1^+^ Tfh cells. Within each subpopulation the expression of CD154 indicated antigen-specific cell activation, CCR6^-^CXCR3^-^ expressing cells were defined as Th2 or Th2-like cells; CCR6^+^CXCR3^-^ as Th17 or Th17-like cells and CCR6^-^CXCR3^+^ as Th1 or Th1-like cells. CD3^-^CD14^-^CD19^+^ cells were selected for the analysis of B cell subsets: CD19^+^CD10^+^ immature cells; CD27^+^CD21^+^ classical memory cells; CD27^-^CD21^+^ naïve cells; CD27^+^CD21^-^ activated memory B cells; CD27^-^CD21^-^ atypical memory B cells; CD20^-^CD21^-^ plasma cells; CD20^+^CD21^+^CD38^+^ activated plasma cells, as previously described [24,25]. FlowJo X and GraphPad PrismV5.0 (GraphPad-Software) were used for data analysis and graphic presentation.

### Quantification of cytokines

IL-21 levels were assessed in plasma and supernatants of cell cultures by Human IL-21 ELISA Reading-Set-Go! kit (2^nd^ Generation) (eBioscience) according to the manufacturer’s instructions and analyzed with the software Softmax. IFN-*γ*, IL-6, IL-10, IL-17 levels were assessed in the same samples using the BD Cytometric Bead Array Human Th1/Th2/Th17 and Human Inflammatory Kits according to the manufacture’s instructions. Samples were acquired using BD FACSVerse system with the BD FACSuite software, analyzed by FCAP Array software and GraphPad Prism.

### Cell purification

After PBMC preparation CD14^+^ monocytes, CD66b^+^ neutrophils and lymphocytes were FACS-sorted using a FACSAria II (BD Biosciences). A second round of sorting was performed to further purify lymphocytes into CD19^+^CD21^+^CD27^-^ B naïve cells, CD19^+^CD27^+^ memory B cells and CD4^+^ T cells. The latter subpopulation was further sorted into CD4^+^CD45RO^-^ naïve T cells, CD4^+^CD45RO^+^CXCR5^-^ memory T cells and CD4^+^CD45RO^+^CXCR5^+^ Tfh cells. The frequency of PD1 and ICOS expressing Tfh cells was assessed in Tfh cells. Anti-CD66b was used to exclude contamination by neutrophils. Purity of sorted cells was >95%. Antibodies used to sort T, B cell subpopulations, monocytes are described in the item Immunophenotyping and intracellular cytokine assessment.

### Reticulocyte purification

The red blood cell pellet from the Ficoll-Hypaque density gradient centrifugation was harvested and washed three times and then resuspended in RPMI to a final hematocrit of 10%. Five milliliters of this suspension was overlaid on 5mL of a 45% Percoll (Sigma Aldrich) solution in a 15mL tube. After centrifugation, floating mature Pv-reticulocytes (Pv-Ret) were collected, washed three times and then resuspended in 1mL RPMI [27].

### Culture of T and B cell subsets

Purified subpopulations were cultivated for 5 and 9 days for assessment of cytokine and immunoglobulin levels. The cultures were performed in RPMI supplemented with penicillin (50U/mL), streptomycin (50ug/mL) and 10% FBS, with stimulation using SEB (1 μg/mL) or Pv-Ret (ratio of 1:1, Pv-Ret:monocytes). 2x10^4^ of naïve T cells or memory T cells or Tfh cells were added to each well alongside the same number of naïve B cells or memory B cells (ratio of 1:1) in the presence of autologous sera (5%) and CD14^+^ cells were added as antigen presenting cells. (20% of total of cells).

### Real-time quantitative PCR

Identification of the *Plasmodium* species (*P*. *vivax*, *P*. *falciparum* and *P*. *ovale*) was done by nested PCR using 0.2mL of peripheral blood that targets variant sequences in the small subunit rRNA gene. The real-time PCR mix was prepared with Syber green PCR master mix (Life Technologies), *P*. *vivax* species-specific primers (75nM) and DNA from blood samples. The real-time PCR was performed in an ABI Prism system 7500 (Applied Biosystems, Foster City, CA) as follows: 95°C for 10 min, 40 cycles of 95°C for 15s, 60°C for 1min.

### Statistical analysis

Statistical analysis was performed using GraphPad Prism V5.0. Differences were considered statistically significant when p≤0.05. Because of the complexity of the experiments performed with purified T and B cell subsets that were present at very low frequencies in the circulation, a p<0.10 is also reported for appreciation. Results were analyzed using a two-tailed paired t-test. Wilcoxon’s test was used when paired samples data did not fit a Gaussian distribution.

Radar graphs were used to analyze the overall immune response taking to account the number of malaria episodes of each patient. The percentage of high producers was calculated for each parameter to create an overall signature. The inner circle represents the 50^th^ percentile, which was taken as threshold to segregate higher and lower expression/production based on the median. Microsoft Excel Software was used for creating radar graphs.

Cytoscape V3.2.0, an open access software, was used for integrating the multiple parameters assessed in the study. Networks were built for each group of patients segregated based on the number of malaria episodes. Correlation analysis was performed using Spearman’s (GraphPad PrismV5.0). Lines were drawn to connect and show associations between attributes, classified as positive (solid line) or negative (dashed line). Lines are displayed with distinct thickness, representing the correlation scores, categorized as strong positive (r ≥ 0.68; thick black line), moderate positive (0.36 ≤ r < 0.68; thin black line), strong negative (r ≤ -0.68; thick gray dashed line), moderate negative (-0.68 < r ≤-0.36; thin gray equal dashed line).

## Supporting information

S1 Fig Tfh are the main sources of IL-21 and induce immunoglobulin secretion by naïve B cells. **A.** Representative dot plots showing sorting strategy used for isolating CD4^+^ T cells and CD19^+^ B cells (first sort). CD19^+^ B cells were stratified based on the expression of CD21 and CD27 molecules to further purify in naïve (CD19^+^CD21^+^) and memory B cells (CD19^+^CD21^+^CD27^+^) (second sort, bottom panel). CD4^+^ T cell subsets were purified based on the expression of CD45RO and CXCR5: naïve (CD4^+^CD45RO-CXCR5-), memory T cells (CD4^+^CD45RO^+^CXCR5-) and Tfh (CD4^+^CD45RO^+^CXCR5^+^) (third sort, top panel). **B.** IL-21 levels measured in supernatant of five and nine days cultures of distinct B and T cell subsets with or without SEB. **C.** IgG levels measured in supernatant of nine days cultures of distinct B and T cell subsets with medium *P*. *vivax*-infected reticulocytes and SEB. *p* values are depicted in the figure.
(TIF)

S2 Fig Immunoglobulin and cell subsets from patients were assessed after treatment. **A-C.** Components of humoral response were measured in patients infected for the first time (black squares) or with multiple infections (blue squares) with *P*. *vivax*. **A.** Pv AMA-1 and PvMSP-1_19_ IgM and IgG were measure in plasma. **B.** Scattered plots showing frequency of activated memory (CD27^+^CD21^-^), classical memory (CD27^+^CD21^+^), naïve (CD27^-^CD21^+^) and atypical memory (CD27^-^CD21^-^) B cells in patients after treatment described above. **C.** Scattered plots showing the proportion of plasma cells (CD21^-^CD20^-^) and IgG, CD38, Ki67, PD-1, IgG and PD-1 and Ki67 and CD38 expressing plasma cells from patients after treatment. All the B cell subsets were analyzed after gating on live CD19^+^ cells. **D.** Frequency of Tfh cells (PD-1^+^ICOS^+^CXCR5^+^CD45RO^+^CD4^+^CD3^+^) cells are shown in patients infected for the first time (black squares) or infected 2 to 5 times (grey squares) or more than 5 times (blue squares) with *P*. *vivax* after treatment. Lines represent median values of the given measurement in each group. *p* values are depicted in the figure.
(TIF)

S3 Fig Biomarker networks of cell subsets, cytokines and antibodies from *P*. *vivax*-infected patients undergoing distinct number of infections. Correlation analyses was performed using Spearman’s (GraphPad PrismV5.0) and between each parameter analyzed and distributed in B-cells subsets, plasma cell subsets, cytokines and antibodies. Circular layouts represent distribution of nodes for parameters assessed in *P*. *vivax*-infected patients (upper circle) and the same patients segregated in first malaria (lower, left circle), 2–5 malaria episodes (lower, middle circle) and more than 5 malaria episodes (right, left circle). Lines connect each two attribute and are classified as positive (solid line) or negative (dashed line). Distinct thickness represents the correlation scores: strong positive (thick line; r ≥ 0.68), moderate positive (thin line; 0.36 ≤ r < 0.68;), strong negative (thick line; r ≤ -0.68;), moderate negative (thin line; -0.68 < r ≤-0.36).
(TIFF)

S4 Fig Representative dot plots of the cell populations and memory and activation markers from a single healthy donor. PBMC from HD were analyzed ex vivo. All the T cell subsets were analyzed after gating on live CD3^+^ cells. **A**. Representative density plots showing frequency of CD4^+^, CD45RO^+^CD4^+^, and CXCR5^+^CD45RO^+^CD4^+^ T cells (left to right) from a single HD (top panel). Representative density plots showing frequency of ICOS, CD40L and PD-1 (left to right) expressing CD4^+^, CD45RO^+^CD4^+^, and CXCR5^+^ CD4^+^ T cells from a single HD (middle and bottom panels). **B**. Gating strategy for the analysis of Tfh cells based on the simultaneous expression of PD-1^+^ICOS^+^CXCR5^+^CD45RO^+^CD4^+^CD3^+^ within live PBMC (density plots) from a single HD. **C**. Representative density plots showing frequency of Tfh cells expressing or not CXCR3 and CCR6 from a single HD. **D**. Representative density plots showing frequency of total B cells (CD19^+^ cells), immature (CD10^+^) B cells, activated memory (CD27^+^CD21^-^), classical memory (CD27^+^CD21^+^), atypical memory (CD27^-^CD21^-^) and naïve (CD27^-^CD21^+^) B cells and plasma cells (CD21^-^CD20^-^) from a single HD.
(TIF)

S5 Fig Tfh cells from patients acutely infected with *P*. *vivax* are the main sources of IL-21. PBMC from healthy donors (HD) and malaria patients before treatment (BT) were cultured with aCD3/CD28 for 8 hours with aCD3/CD28 and IL-21 production by Tfh cells analyzed by flow cytometry. p value is depicted in the figure.
(TIF)

S6 Fig Increase in the reactivity index of IgM against AMA-1 from *Plasmodium vivax* during malaria. **A.** *Plasmodium vivax*-specific antibodies were measured by enzyme-linked immunosorbent assay (ELISA). PvAMA-1 IgM and IgG were measured in plasma of patients during acute malaria (BT) and after treatment (AT). **B.** IgG subclasses against PvAMA-1 were measure in plasma of patients during acute malaria (BT) and after treatment (AT). Lines represent median values of the given measurement in each group. Dotted lines represent healthy donors. *p* values are depicted in the figure.
(TIF)

S1 Table Clinical characteristic and laboratory data. (DOCX)

S2 Table Antibodies used for flow cytometry and ELISA: Immunoglobulin levels, immunophenotyping, cell sorting and functional experiments. (DOCX)

S3 Table Frequencies of T and B cell subsets. (DOCX)

S4 Table Frequencies of cell subsets segregated by malaria episodes. (DOCX)
