# Supplementary figures and images for: T follicular helper cells regulate the activation of B lymphocytes and antibody production during Plasmodium vivax infection
Source: PLoS Pathog. 2017 Jul 10;13(7):e1006484. doi: 10.1371/journal.ppat.1006484 (PMC5519210; doi:10.1371/journal.ppat.1006484)

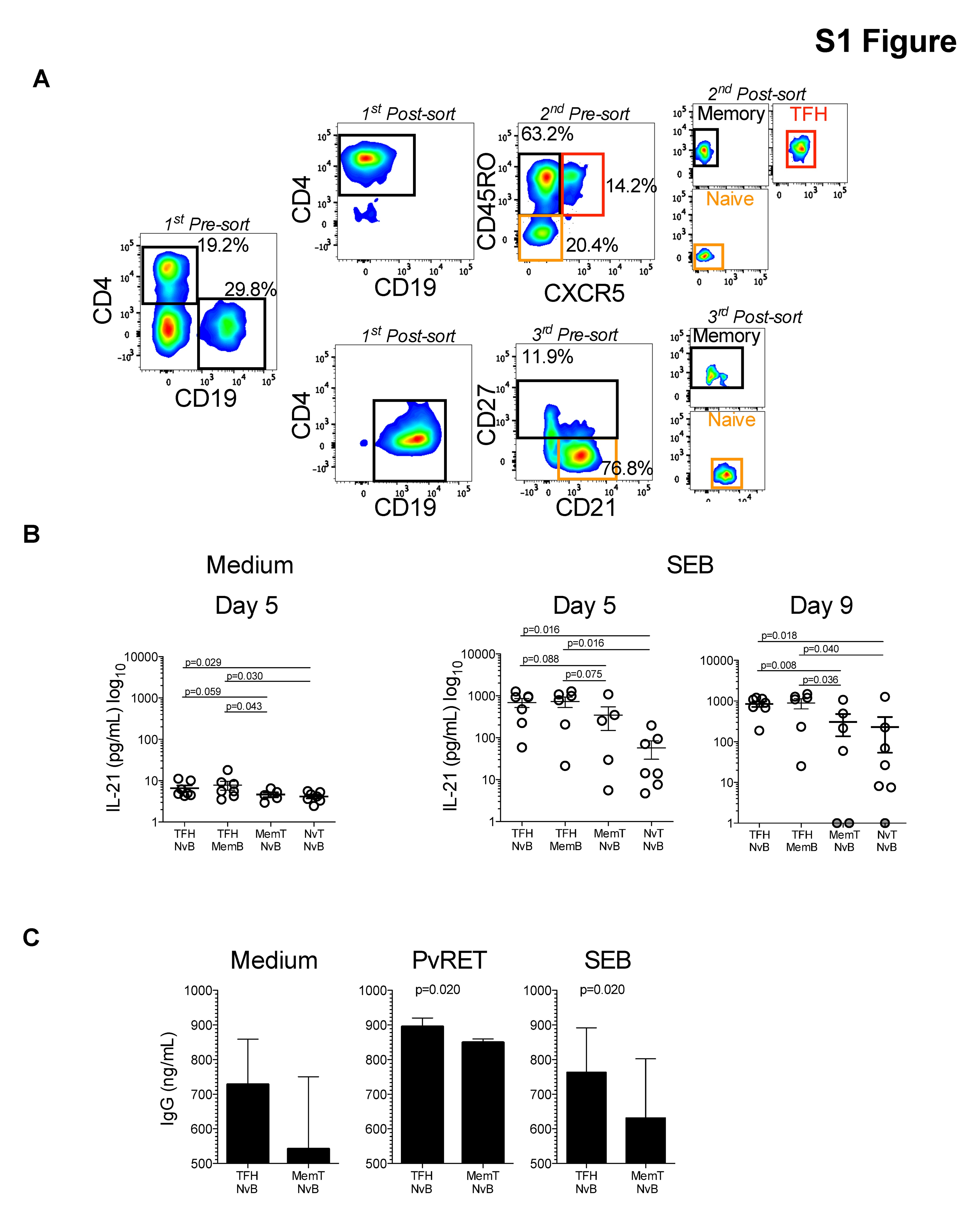

Supplement: S1 Fig — A. Representative dot plots showing sorting strategy used for isolating CD4+ T cells and CD19+ B cells (first sort). CD19+ B cells were stratified based on the expression of CD21 and CD27 molecules to further purify in naïve (CD19+CD21+) and memory B cells (CD19+CD21+CD27+) (second sort, bottom panel). CD4+ T cell subsets were purified based on the expression of CD45RO and CXCR5: naïve (CD4+CD45RO-CXCR5-), memory T cells (CD4+CD45RO+CXCR5-) and Tfh (CD4+CD45RO+CXCR5+) (third sort, top panel). B. IL-21 levels measured in supernatant of five and nine days cultures of distinct B and T cell subsets with or without SEB. C. IgG levels measured in supernatant of nine days cultures of distinct B and T cell subsets with medium P. vivax-infected reticulocytes and SEB. p values are depicted in the figure. (TIF) [file ppat.1006484.s001.tif]

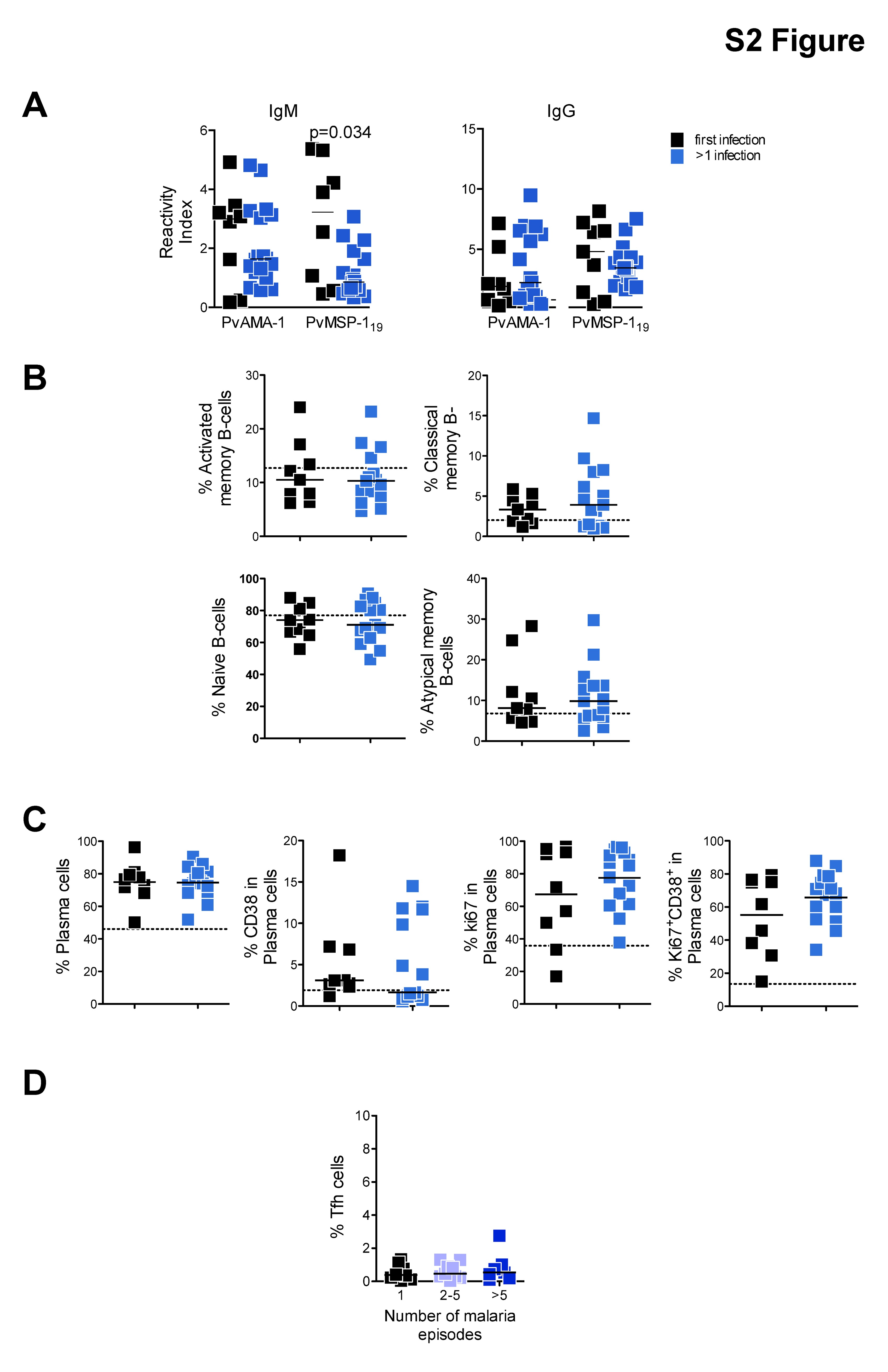

Supplement: S2 Fig — A-C. Components of humoral response were measured in patients infected for the first time (black squares) or with multiple infections (blue squares) with P. vivax. A. Pv AMA-1 and PvMSP-119 IgM and IgG were measure in plasma. B. Scattered plots showing frequency of activated memory (CD27+CD21-), classical memory (CD27+CD21+), naïve (CD27-CD21+) and atypical memory (CD27-CD21-) B cells in patients after treatment described above. C. Scattered plots showing the proportion of plasma cells (CD21-CD20-) and IgG, CD38, Ki67, PD-1, IgG and PD-1 and Ki67 and CD38 expressing plasma cells from patients after treatment. All the B cell subsets were analyzed after gating on live CD19+ cells. D. Frequency of Tfh cells (PD-1+ICOS+CXCR5+CD45RO+CD4+CD3+) cells are shown in patients infected for the first time (black squares) or infected 2 to 5 times (grey squares) or more than 5 times (blue squares) with P. vivax after treatment. Lines represent median values of the given measurement in each group. p values are depicted in the figure. (TIF) [file ppat.1006484.s002.tif]

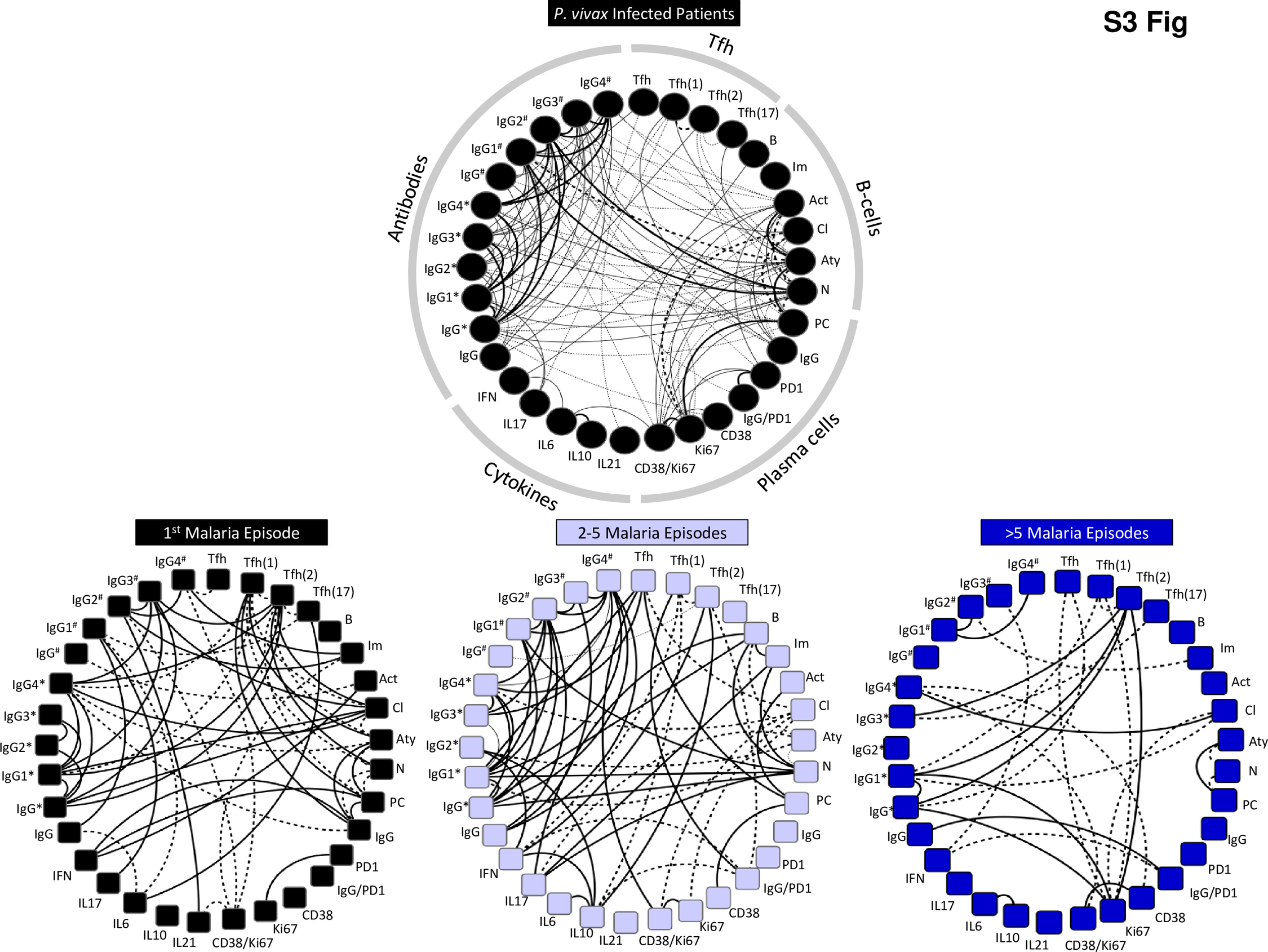

Supplement: S3 Fig — Correlation analyses was performed using Spearman’s (GraphPad PrismV5.0) and between each parameter analyzed and distributed in B-cells subsets, plasma cell subsets, cytokines and antibodies. Circular layouts represent distribution of nodes for parameters assessed in P. vivax-infected patients (upper circle) and the same patients segregated in first malaria (lower, left circle), 2–5 malaria episodes (lower, middle circle) and more than 5 malaria episodes (right, left circle). Lines connect each two attribute and are classified as positive (solid line) or negative (dashed line). Distinct thickness represents the correlation scores: strong positive (thick line; r ≥ 0.68), moderate positive (thin line; 0.36 ≤ r < 0.68;), strong negative (thick line; r ≤ -0.68;), moderate negative (thin line; -0.68 < r ≤-0.36). (TIFF) [file ppat.1006484.s003.tiff]

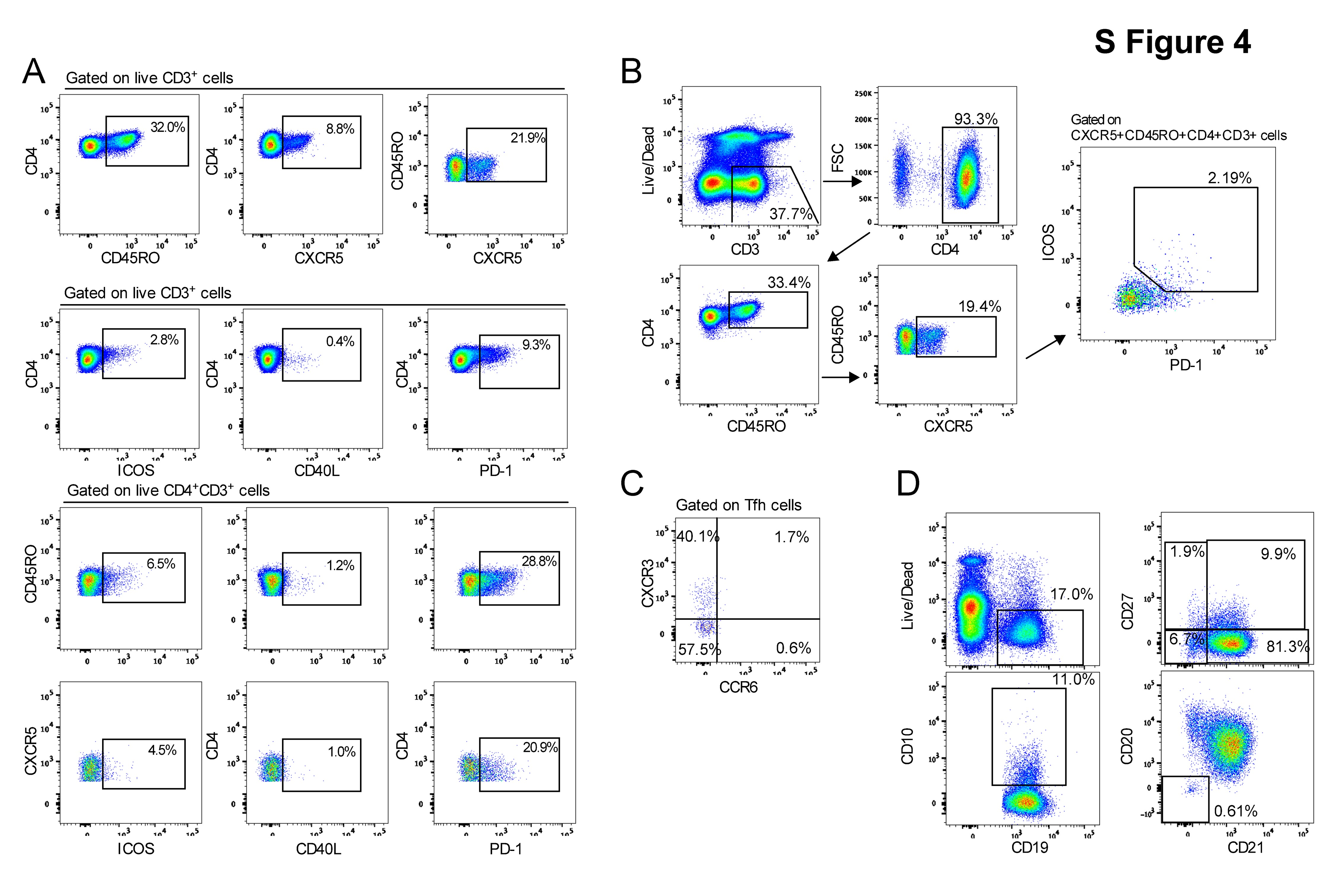

Supplement: S4 Fig — PBMC from HD were analyzed ex vivo. All the T cell subsets were analyzed after gating on live CD3+ cells. A. Representative density plots showing frequency of CD4+, CD45RO+CD4+, and CXCR5+CD45RO+CD4+ T cells (left to right) from a single HD (top panel). Representative density plots showing frequency of ICOS, CD40L and PD-1 (left to right) expressing CD4+, CD45RO+CD4+, and CXCR5+ CD4+ T cells from a single HD (middle and bottom panels). B. Gating strategy for the analysis of Tfh cells based on the simultaneous expression of PD-1+ICOS+CXCR5+CD45RO+CD4+CD3+ within live PBMC (density plots) from a single HD. C. Representative density plots showing frequency of Tfh cells expressing or not CXCR3 and CCR6 from a single HD. D. Representative density plots showing frequency of total B cells (CD19+ cells), immature (CD10+) B cells, activated memory (CD27+CD21-), classical memory (CD27+CD21+), atypical memory (CD27-CD21-) and naïve (CD27-CD21+) B cells and plasma cells (CD21-CD20-) from a single HD. (TIF) [file ppat.1006484.s004.tif]

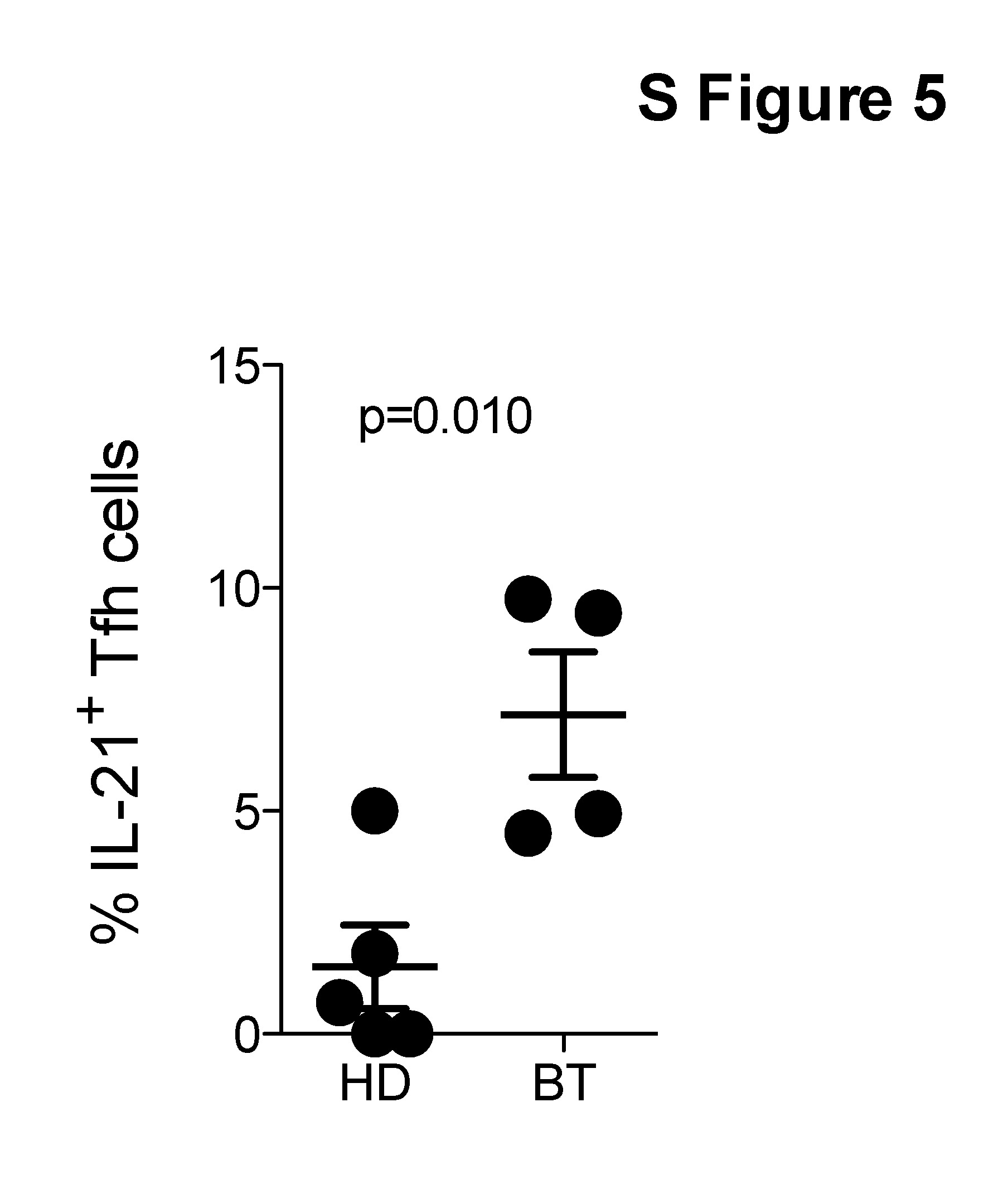

Supplement: S5 Fig — PBMC from healthy donors (HD) and malaria patients before treatment (BT) were cultured with aCD3/CD28 for 8 hours with aCD3/CD28 and IL-21 production by Tfh cells analyzed by flow cytometry. p value is depicted in the figure. (TIF) [file ppat.1006484.s005.tif]

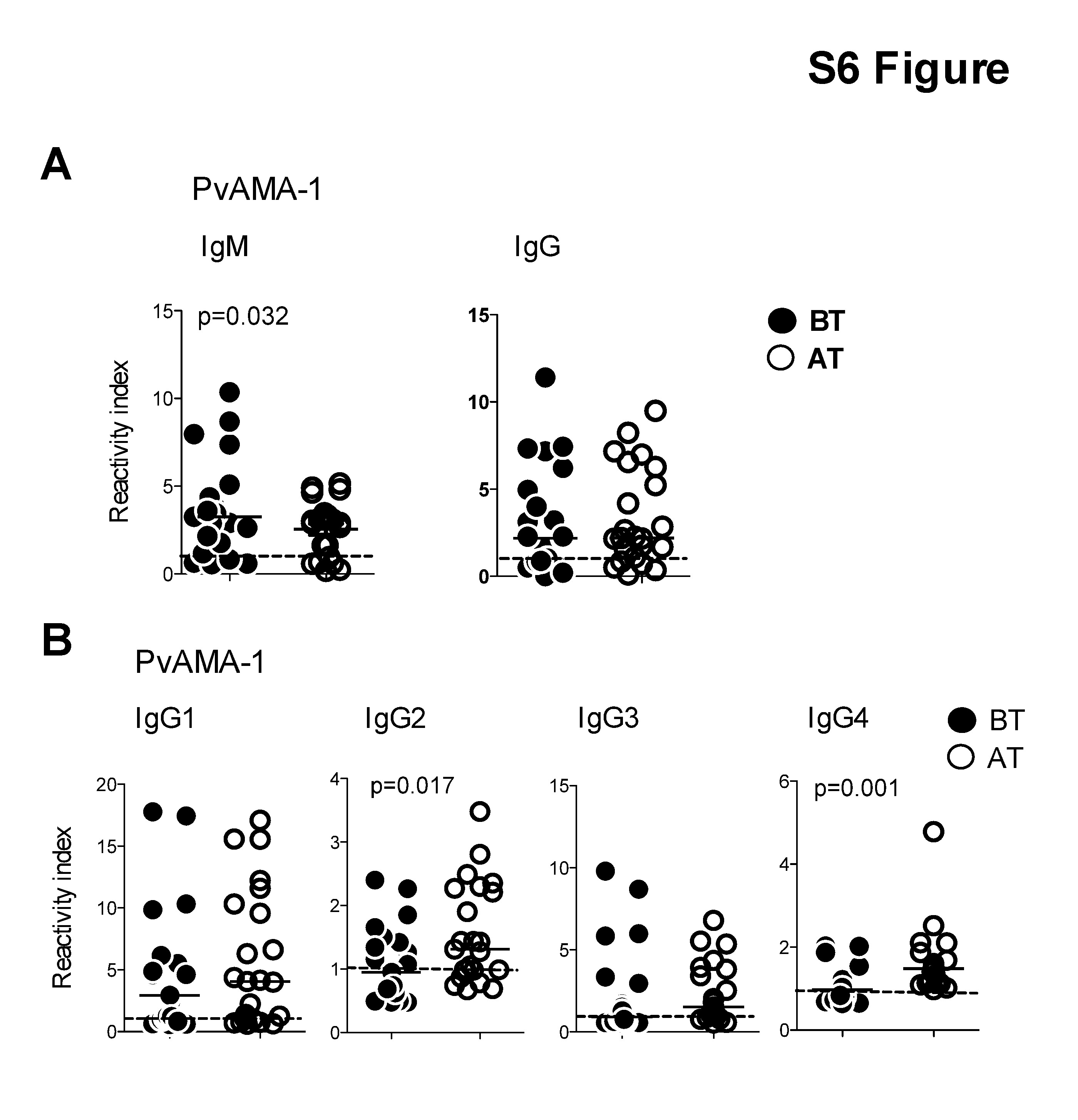

Supplement: S6 Fig — A. Plasmodium vivax-specific antibodies were measured by enzyme-linked immunosorbent assay (ELISA). PvAMA-1 IgM and IgG were measured in plasma of patients during acute malaria (BT) and after treatment (AT). B. IgG subclasses against PvAMA-1 were measure in plasma of patients during acute malaria (BT) and after treatment (AT). Lines represent median values of the given measurement in each group. Dotted lines represent healthy donors. p values are depicted in the figure. (TIF) [file ppat.1006484.s006.tif]
