# Supplementary material for: T follicular helper cells regulate the activation of B lymphocytes and antibody production during Plasmodium vivax infection
Source: PLoS Pathog. 2017 Jul 10;13(7):e1006484. doi: 10.1371/journal.ppat.1006484 (PMC5519210; doi:10.1371/journal.ppat.1006484)
Supplement: S1 Table — (DOCX) [file ppat.1006484.s007.docx]

S1 Table. Clinical characteristic and laboratory data

| **Patient ID** | **Gender** | **Age** | **# Malaria Episodes** | | | **Parasites/μl of blood and qPCR** | | | | | **Hematological Records** | | | | | | | | **Symptoms** | | |
| --- | --- | --- | --- | --- | --- | --- | --- | --- | --- | --- | --- | --- | --- | --- | --- | --- | --- | --- | --- | --- | --- |
|  |  |  | 1st | <5 | ≥5 | *≤ 500* | | *500 - 10000* | *> 10000* | qPCR | *Hemoglobin*  *g/dL.* | *Hematocrit*  *%* | *Red Blood Cells10^6^/mm^3^* | *White Blood Cells 10^3^/mm^3^* | *Platelets 10^3^/mm^3^* | *Creatinine mg/dL* | *AST*  *U/L* | *ALT U/L* | Myalgia | Headache | Chills |
| Tfh1 BT* | Male | 28 |  |  | X |  | | X |  | 1,622.46 | 14.4 | 43.7 | 4.98 | 4.7 | 41,000 | 0.30 | 39 | 31 | Yes | No | Yes |
| Tfh1 AT** |  |  |  |  |  | ND*** | | | |  | 15.5 | 46.9 | 5.54 | 6.5 | 177,000 | 0.49 | 40 | 38 | No | | |
| Tfh2 BT | Male | 34 |  |  | X | X | |  |  | 57.82 | 13.1 | 37.6 | 4.31 | 5.1 | 151,000 | 0.60 | 11 | 15 | Yes | Yes | Yes |
| Tfh2 AT |  |  |  |  |  | ND | | | |  | 13.6 | 39.1 | 4.51 | 8.0 | 249,000 | 0.90 | 16 | 15 | No | | |
| Tfh3 BT | Male | 38 | X |  |  |  | | X |  | 1,785.76 | 13.6 | 41.4 | 5.24 | 4.0 | 52,000 | 0.83 | 85 | 68 | Yes | Yes | Yes |
| Tfh3 AT |  |  |  |  |  | ND | | | |  | 15.9 | 47.7 | 5.82 | 6.9 | 238,000 | 0.40 | 14 | 15 | No | | |
| Tfh4 BT | Male | 45 |  | X |  |  | | X |  | 12,641.45 | 13.3 | 41.9 | 4.42 | 5.1 | 65,000 | 1.00 | 64 | 35 | Yes | Yes | Yes |
| Tfh4 AT |  |  |  |  |  | ND | | | |  | 17.4 | 50.6 | 5.35 | 4.9 | 195,000 | 0.90 | 23 | 42 | No | | |
| Tfh5 BT | Male | 37 | X |  |  | X | |  |  | 846.69 | 13.6 | 39.1 | 4.84 | 2.5 | 30,000 | 1.09 | 172 | 178 | Yes | Yes | Yes |
| Tfh5 AT |  |  |  |  |  | ND | | | |  | 14.5 | 43.1 | 5.20 | 4.7 | 94,000 | NA**^+^** | 25 | 21 | No | | |
| Tfh6 BT | Male | 32 |  |  | X | X | |  |  | 2.24 | 15.0 | 44.0 | 5.91 | 5.8 | 160,000 | 0.88 | 32 | 39 | No | Yes | Yes |
| Tfh6 AT |  |  |  |  |  | ND | | | |  | 16.3 | 43.0 | 5.70 | 6.6 | 130,000 | 1.20 | 18 | 37 | No | | |
| Tfh7 BT | Male | 41 |  | X |  | X | |  |  | 71.70 | 14.3 | 43.9 | 5.35 | 9.8 | 20,700 | 1.10 | 20 | 29 | Yes | Yes | No |
| Tfh7 AT |  |  |  |  |  | ND | | | |  | 14.9 | 45.6 | 5.33 | 7.6 | 27,600 | 0.90 | 23 | 38 | No | | |
| Tfh8 BT | Male | 27 | X |  |  |  | | X |  | 52.94 | NA | NA | NA | NA | NA | 0.90 | 82 | 43 | Yes | Yes | No |
| Tfh8 AT |  |  |  |  |  | ND | | | |  | 15.3 | 43.5 | 4.90 | 11.4 | 2,300 | 0.80 | 20 | 13 | No | | |
| Tfh9 BT | Female | 33 | X |  |  | X | |  |  | 8.85 | 13.3 | 39.1 | 4.65 | 4.5 | 84,000 | 0.50 | 247 | 147 | Yes | Yes | No |
| Tfh9 AT |  |  |  |  |  | ND | | | |  | NA | NA | NA | NA | NA | 0.40 | 21 | 14 | No | | |
| Tfh10 BT | Male | 40 |  |  | X |  | | X |  | 307.21 | 16.1 | 48.9 | 6.09 | 5.9 | 93,000 | 0.80 | 34 | 41 | Yes | Yes | No |
| Tfh10 AT |  |  |  |  |  | ND | | | |  | 13.9 | 40.9 | 5.07 | 5.4 | 22,600 | 1.00 | 19 | 24 | No | | |
| Tfh11 BT | Male | 40 |  |  | X |  | |  | X | 2,737.50 | 13.6 | 40.5 | NA | 5.2 | 157,000 | 0.80 | 23 | 16 | Yes | No | No |
| Tfh11 AT |  |  |  |  |  | ND | | | |  | 13.7 | 41.0 | 4.16 | 4.7 | 47,800 | 0.70 | 24 | 20 | No | | |
| Tfh12 BT | Male | 27 |  |  | X |  | | X |  | 20.61 | 14.5 | 44.0 | ND | 5.3 | 187,000 | 0.80 | 27 | 34 | Yes | Yes | Yes |
| Tfh12 AT |  |  |  |  |  | ND | | | |  | 14.5 | 44.0 | 4.92 | 5.3 | 187,000 | 0.80 | 27 | 34 | No | | |
| Tfh13 BT | Male | 41 |  | X |  |  | | X |  | 775.75 | 12.1 | 35.4 | 4.05 | 5.8 | 71,000 | 1.00 | 18 | 11 | Yes | Yes | No |
| Tfh13 AT |  |  |  |  |  | ND | | | |  | 14.4 | 41.6 | 4.71 | 1.1 | 248,000 | 1.00 | 20 | 12 | No | | |
| Tfh14 BT | Male | 27 |  | X |  |  | | X |  | ND | 14.4 | 41.8 | 4.76 | 4.3 | 14,800 | 1.00 | 32 | 34 | Yes | No | No |
| Tfh14 AT |  |  |  |  |  | ND | | | |  | 15.8 | 45.4 | 5.17 | 6.8 | 250,000 | 0.80 | 22 | 26 | No | | |
| Tfh15 BT | Male | 22 |  |  | NI**** |  | | X |  | ND | 14.1 | 40.0 | 4.69 | 5.6 | 234,000 | 0.80 | 22 | 28 | Yes | Yes | No |
| Tfh15 AT |  |  |  |  |  | ND | | | |  | 14.1 | 40.0 | 4.69 | 5.6 | 234,000 | 0.80 | 22 | 28 | No | | |
| Tfh16 BT | Male | 22 |  |  | X |  | | X |  | ND | 14.1 | 41.3 | 4.86 | 3.9 | 78,000 | 0.70 | 20 | 15 | Yes | Yes | Yes |
| Tfh16 AT |  |  |  |  |  | ND | | | |  | 12.4 | 40.1 | 4.61 | 4.3 | 109,000 | 0.94 | 16 | 13.7 | No | | |
| Tfh17 BT | Male | 28 |  |  | NI |  | | X |  | ND | 15.8 | 43.9 | 5.17 | 8.3 | 134,000 | 0.80 | 18 | 16 | Yes | No | No |
| Tfh17 AT |  |  |  |  |  | ND | | | |  | NA | NA | NA | NA | NA | NA | NA | NA | No | | |
| Tfh18 BT | Male | 29 | X |  |  |  | | X |  | 8,555.48 | 13.1 | 38.7 | 4.48 | 4.8 | ND | 0.80 | 41 | 100 | Yes | Yes | Yes |
| Tfh18 AT |  |  |  |  |  | ND | | | |  | NA | NA | NA | NA | NA | 0.70 | 25 | 83 | No | | |
| Tfh19 BT | Male | 56 |  | X |  |  | | X |  | 586.66 | 14.7 | 43.0 | 4.87 | 5.0 | 210,000 | 0.50 | 26 | 15 | Yes | Yes | Yes |
| Tfh19 AT |  |  |  |  |  | ND | | | |  | 12.2 | 40.6 | 4.48 | 7.0 | 340,000 | 0.60 | 20 | 13 | No | | |
| Tfh20 BT | Female | 27 |  | X |  |  | | X |  | 2,578.90 | 11.6 | 36.2 | 4.44 | 5.0 | 135,000 | 0.60 | 26 | 22 | Yes | Yes | Yes |
| Tfh20 AT |  |  |  |  |  | ND | | | |  | 11.3 | 36.7 | 4.56 | 8.9 | 393,000 | 0.90 | 21 | 16 | No | | |
| Tfh21 BT | Male | 18 |  | X |  |  | | X |  | 244.89 | 13.2 | 37.7 | 4.40 | 7.5 | 121,000 | 1.00 | 49 | 26 | Yes | Yes | Yes |
| Tfh21 AT |  |  |  |  |  | ND | | | |  | 13.5 | 40.9 | 4.68 | 7.0 | 159,000 | 1.00 | 26 | 23 | No | | |
| Tfh22 BT | Male | 38 |  | X |  |  | | X |  | 1,760.63 | 13.8 | 41.3 | 4.37 | 2.9 | 70,000 | 0.90 | 22 | 9 | Yes | Yes | Yes |
| Tfh22 AT |  |  |  |  |  | ND | | | |  | NA | NA | NA | NA | NA | NA | NA | NA | No | | |
| Tfh23 BT | Male | 43 | X |  |  |  | X | |  | 275.67 | 13.6 | 39.8 | 4.61 | 2.1 | 34,000 | 0.80 | 48 | 43 | Yes | Yes | Yes |
| Tfh23 AT |  |  |  |  |  | ND | | | |  | 14.1 | 44.0 | 5.00 | 5.9 | 250,000 | 0.70 | 21 | 16 | No | | |

*Before treatment /**After treatment/***Not detected by blood smear and PCR/****NI (Not informed)/ ^+^Not assessed (NA)
