# Supplementary material for: T follicular helper cells regulate the activation of B lymphocytes and antibody production during Plasmodium vivax infection
Source: PLoS Pathog. 2017 Jul 10;13(7):e1006484. doi: 10.1371/journal.ppat.1006484 (PMC5519210; doi:10.1371/journal.ppat.1006484)
Supplement: S2 Table — (DOCX) [file ppat.1006484.s008.docx]

S2 Table. Antibodies used for flow cytometry and ELISA: immunoglobulin levels, immunophenotyping, cell sorting and functional experiments

| **Antibodies and streptavidin** | **Fuorochromes** | **Company** | **Clone** |
| --- | --- | --- | --- |
| BCL6 | V450 | BD | K112-91 |
| CCR6 | PERCP eFluor 710 | eBioscience | R6H1 |
| CD3 | eFluor 450 | eBioscience | OKT3 |
| CD3 | Qdot 655 | Invitrogen | S4.1 |
| CD4 | Qdot 605 | Invitrogen | S3.5 |
| CD10 | Biotinylated | eBioscience | SN5c |
| CD14 | eFluor 450 | eBioscience | 61D3 |
| CD14 | APC | eBioscience | 61D3 |
| CD19 | PECy7 | eBioscience | SJ25C1 |
| CD20 | Qdot 655 | Invitrogen | Q10305 |
| CD21 | FITC | eBioscience | HB5 |
| CD27 | APC H7 | BD | M7271 |
| CD38 | PE | BD | HIT2 |
| CD45RO | Alexa 700 | BD | UCHL1 |
| CD66b | PE | Biolegend | G10F5 |
| CD154 | APC-eFluor 780 | eBioscience | 24-31 |
| CXCR3 | PE | BD | 557182 |
| CXCR5 | Alexa Fluor 488 | BD | RF8B2 |
| ICOS | PE-Cy7 | eBioscience | ISA-3 |
| IgG | Alexa 700 | BD | G18-145 |
| IL-21 | eFluor 660 | eBioscience | 3ª3-N2 |
| Live/Dead | Acqua | Invitrogen | L34957 |
| Live/Dead | Violet | Invitrogen | L34955 |
| Ki67 | PERCPCy5.5 | BD | B56 |
| PD-1 | APC | Biolegend | EH12.2H7 |
| Streptoavidin | Qdot 605 | Invitrogen | Q10101MP |
| IgG | - | SIGMA | Polyclonal |
| IgM | - | SIGMA | Polyclonal |
| IgG1 | - | SIGMA | 8c/6-39 |
| IgG2 | - | SIGMA | HP-6014 |
| IgG3 | - | SIGMA | HP-6050 |
| IgG4 | - | SIGMA | HP-6025 |
