# Supplementary material for: T follicular helper cells regulate the activation of B lymphocytes and antibody production during Plasmodium vivax infection
Source: PLoS Pathog. 2017 Jul 10;13(7):e1006484. doi: 10.1371/journal.ppat.1006484 (PMC5519210; doi:10.1371/journal.ppat.1006484)
Supplement: S3 Table — (DOCX) [file ppat.1006484.s009.docx]

S3 Table. Frequencies of T and B cell subsets

|  | Patient and controls | | | |
| --- | --- | --- | --- | --- |
|  | Cell (%) | BT | AT | HD |
| Gated on live CD3^+^ cells | CD45RO in CD4 T cells | 63.75; 19 | 57.45; 17.5 | 64.95; 28.30 |
|  | CXCR5 in CD4 T cells | 11.85; 6.10 | 12.65; 4.60 | 14.05; 7.42 |
|  | CXCR5 in CD45RO T cells | 17.20; 9.65 | 20.15; 9.60 | 21.20; 2.30 |
|  | ICOS in CD4 T cells | 12.80; 13.39* | 7.13; 4.50 | 4.68; 3.16 |
|  | CD40L in CD4 T cells | 0.60; 0.59 | 0.38; 0.25 | 0.49; 0.28 |
|  | PD1 in CD4 T cells | 22.55; 7.62 | 16.60; 10.23 | 20.75; 10.51 |
| Gated on live CD4^+^CD3^+^ cells | ICOS in CD45RO T cells | 23.45; 14.3* | 10.25; 7.57 | 7.51; 1.75 |
|  | CD40L in CD45RO T cells | 2.19; 1.88 | 1.10; 1.18 | 1.58; 0.86 |
|  | PD1 in CD45RO T cells | 35.60; 13.48 | 26.05; 11.25 | 31.80; 14.02 |
|  | ICOS in CXCR5 T cells | 15.05; 15.55* | 8.47; 9.9 | 5.01; 3.60 |
|  | CD40L in CXCR5 T cells | 2.18; 1.82 | 1.74; 0.68 | 1.54; 0.85 |
|  | PD1 in CXCR5 T cells | 26.50; 10.93 | 22.20; 14.73 | 26.55; 10.90 |
| Gated on CXCR5^+^CD45RO^+^CD4^+^CD3^+^ cells | Tfh (PD-1^+^ICOS^+^ cells) |  |  |  |
|  |  | 1.24; 1.04* | 0.45; 0.78 | 0.33; 0.38 |
|  |  |  |  |  |
| Gated on Tfh cells | CXCR3^+^CCR6^-^ | 54.00; 23.12* | 41.35; 19.83 | 31.20; 9.53 |
|  | CXCR3^-^CCR6^-^ | 22.35; 25.35 | 43.75; 16.60 | 45.55; 19.00 |
|  | CXCR3^-^CCR6^+^ | 15.10; 8.04 | 5.51; 4.32 | 6.76; 8.83 |
| Gated on CD45RO^+^CD4^+^CD3^+^ T cells | CXCR3^+^CCR6^-^ | 29.95; 14.25 | 30.15; 12.20 | 35.20; 0.96 |
|  | CXCR3^-^CCR6^-^ | 54.80; 18.97 | 56.45; 17.47 | 50.70; 18.67 |
|  | CXCR3^-^CCR6^+^ | 7.71; 3.42 | 7.52; 4.85 | 7.71; 3.92 |
| Gated on CXCR5^+^CD45RO^+^CD4^+^CD3^+^ Cells | CXCR3^+^CCR6^-^ | 31.85; 19.72 | 38.10; 12.93 | 41.10; 9.20 |
|  | CXCR3^-^CCR6^-^ | 51.15; 29.1 | 55.00; 17.14 | 50.70; 18.67 |
|  | CXCR3^-^CCR6^+^ | 0.89; 1.13* | 0.53; 1.75 | 0.43; 0.80 |
| Gated on live cells | CD19^+^ cells | 12.80; 8.92 | 15.10; 10.61 | 14.55; 3.72 |
| Gated on CD19^+^ cells | Immature B-cells | 11.55; 6.95 | 12.75; 7.85 | 1.58; 0.86 |
|  | Activated memory B-cells | 8.10; 8.94* | 3.63; 4.21 | 2.01; 2.04 |
|  | Classical memory B-cells | 6.31; 6.42* | 10.40; 6.86 | 12.70; 6.29 |
|  | Atypical memory B-cells | 16.30; 26.7* | 9.00; 7.93 | 6.79; 1.88 |
|  | Naive B-cells | 61.30; 30.45 | 72.75; 19.1 | 76.95; 13.00 |
|  | Plasma cells | 18.35; 23.89* | 2.92; 7.98 | 1.95; 1.71 |
|  | IgG in Plasma cells | 17.80; 14.5 | 14.70; 22.23 | 11.50; 12.79 |
|  | CD38 in Plasma cells | 82.76; 9.92* | 74.75; 12.74^#^ | 46.05; 29.73 |
|  | PD-1 in Plasma cells | 33.50; 21.28* | 24.15; 15.60 | 14.70; 10.36 |
|  | Ki67 in Plasma cells | 96.90; 11.48* | 81.50; 35.29^#^ | 35.88; 31.26 |
|  | IgG^+^PD-1^+^ in Plasma cells | 19.50; 17.75 | 8.72; 10.11 | 13.50; 10.70 |
|  | Ki67^+^CD38^+^ in Plasma cells | 78.90; 11.40* | 68.20; 25.10^#^ | 36.00; 29.62 |

*significant differences between HD and BT; ^#^significant differences between HD and AT. HD (Healthy Donor); BT (*P. vivax*-infected patients, Before Treatment); AT (After Treatment). Numbers: Median; IQR.
