# Supplementary material for: T follicular helper cells regulate the activation of B lymphocytes and antibody production during Plasmodium vivax infection
Source: PLoS Pathog. 2017 Jul 10;13(7):e1006484. doi: 10.1371/journal.ppat.1006484 (PMC5519210; doi:10.1371/journal.ppat.1006484)
Supplement: S4 Table — (DOCX) [file ppat.1006484.s010.docx]

S4 Table. Frequencies of cell subsets segregated by malaria episodes

|  | Patients and controls | | | | |
| --- | --- | --- | --- | --- | --- |
|  | Cell (%) | first infection | >1 infection | | HD |
| Before treatment | Activated memory B cells | 15.00; 8.07* | 4.53; 5.93^#^ | | 2.01; 2.04 |
|  | Classical memory B cells | 3.21; 4.77* | 6.66; 6.57^#^ | | 12.70; 6.29 |
|  | Atypical memory B cells | 40.10; 16.7* | 12.20; 16.24^#^ | | 6.79; 1.88 |
|  | Naive B-cells | 29.00; 29.9* | 70.60; 16.5 | | 76.95; 13.00 |
|  | Plasma cells | 57.30; 42.30* | 11.80; 17.38^#^ | | 1.95; 1.71 |
|  | CD38 in Plasma cells | 87.95; 9.63* | 81.42; 11.38^#^ | | 46.05; 29.73 |
|  | Ki67 in Plasma cells | 98.10; 1.48* | 91.97; 11.4^#^ | | 35.88; 31.26 |
|  | Ki67^+^CD38^+^ in Plasma cells | 87.50; 10.00* | 78.50; 8.1^#^ | | 36.00; 29.62 |
| After treatment | Activated memory B cells | 3.34; 3.03 | 3.92; 6.69 | | 2.01; 2.04 |
|  | Classical memory B cells | 10.50; 8.12 | 10.30; 7.30 | | 12.70; 6.29 |
|  | Atypical memory B cells | 8.11; 13.24 | 9.85; 7.75 | | 6.79; 1.88 |
|  | Naive B-cells | 74.00; 17.35 | 71.10; 21.90 | | 76.95; 13.00 |
|  | Plasma cells | 3.10; 4.52 | 1.65; 10.69 | | 1.95; 1.71 |
|  | CD38 in Plasma cells | 76.62; 10.24* | 74.20; 13.00^#^ | | 46.05; 29.73 |
|  | Ki67 in Plasma cells | 71.80; 52.53 | 85.10; 31.72^#^ | | 35.88; 31.26 |
|  | Ki67^+^CD38^+^ in Plasma cells | 61.70; 41.3 | 68.20; 26.10^#^ | | 36.00; 29.62 |
|  |  |  | 2-5 infections | >5 infections |  |
| Before treatment | Tfh | 0.85; 1.03* | 1.10; 1.22^#^ | 1.69; 1.78^#^ | 0.33; 0.38 |
| After treatment | Tfh | 0.38; 0.90 | 0.46; 0.84 | 0.54; 0.83 | 0.33; 0.38 |

*significant differences between HD and first infection; ^#^significant differences between HD and > 1 infection. HD (Healthy Donor). Numbers: Median; IQR.
